# Identifying effective intervention strategies to reduce children’s screen time: a systematic review and meta-analysis

**DOI:** 10.1186/s12966-021-01189-6

**Published:** 2021-09-16

**Authors:** Alexis Jones, Bridget Armstrong, R. Glenn Weaver, Hannah Parker, Lauren von Klinggraeff, M. W. Beets

**Affiliations:** Department of Exercise Science, Arnold School of Public Health, University of South Carolina, Columbia, SC USA

**Keywords:** Screen time, Children, Interventions, Meta-analysis, Behavior change techniques

## Abstract

**Background:**

Excessive screen time ($\ge$ 2 h per day) is associated with childhood overweight and obesity, physical inactivity, increased sedentary time, unfavorable dietary behaviors, and disrupted sleep. Previous reviews suggest intervening on screen time is associated with reductions in screen time and improvements in other obesogenic behaviors. However, it is unclear what study characteristics and behavior change techniques are potential mechanisms underlying the effectiveness of behavioral interventions. The purpose of this meta-analysis was to identify the behavior change techniques and study characteristics associated with effectiveness in behavioral interventions to reduce children’s (0–18 years) screen time.

**Methods:**

A literature search of four databases (Ebscohost, Web of Science, EMBASE, and PubMed) was executed between January and February 2020 and updated during July 2021. Behavioral interventions targeting reductions in children’s (0–18 years) screen time were included. Information on study characteristics (e.g., sample size, duration) and behavior change techniques (e.g., information, goal-setting) were extracted. Data on randomization, allocation concealment, and blinding was extracted and used to assess risk of bias. Meta-regressions were used to explore whether intervention effectiveness was associated with the presence of behavior change techniques and study characteristics.

**Results:**

The search identified 15,529 articles, of which 10,714 were screened for relevancy and 680 were retained for full-text screening. Of these, 204 studies provided quantitative data in the meta-analysis. The overall summary of random effects showed a small, beneficial impact of screen time interventions compared to controls (SDM = 0.116, 95CI 0.08 to 0.15). Inclusion of the Goals, Feedback, and Planning behavioral techniques were associated with a positive impact on intervention effectiveness (SDM = 0.145, 95CI 0.11 to 0.18). Interventions with smaller sample sizes (*n* < 95) delivered over short durations (< 52 weeks) were associated with larger effects compared to studies with larger sample sizes delivered over longer durations. In the presence of the Goals, Feedback, and Planning behavioral techniques, intervention effectiveness diminished as sample size increased.

**Conclusions:**

Both intervention content and context are important to consider when designing interventions to reduce children’s screen time. As interventions are scaled, determining the active ingredients to optimize interventions along the translational continuum will be crucial to maximize reductions in children’s screen time.

**Supplementary Information:**

The online version contains supplementary material available at 10.1186/s12966-021-01189-6.

## Introduction

In the past decade, screen time has become a ubiquitous behavior in the daily lives of children and adolescents worldwide. The Sedentary Behavior Research Network defines screen time as the amount of time spent engaging with screens – such as tablets, computers, or smartphones – while sitting, standing, or being physically active [1]. Between 45–80% of children and adolescents fail to meet international recommendations of < 2 h per day of screen time [2, 3]. According to international 24-h movement guidelines, infants (birth to 1 year old) and toddlers (less than 2 years old) should not engage in sedentary screen time [4]. Further, preschoolers (ages 3–4 years old) should not exceed 1 h of sedentary screen time per day [4]. As children age, screen time guidelines are adjusted to recommend no more than 2 h per day of recreational screen time for children and adolescents (5–17 years old) [5]. Independent of physical activity and sedentary behavior, excess screen time ($\ge$ 2 h per day) is associated with childhood overweight and obesity (OWOB) [6–8]. Excess screen time is also associated with unfavorable obesogenic behaviors, such as physical inactivity, increased sedentary time, unfavorable dietary behaviors, and disrupted sleep [9, 10]. Interventions targeting screen time can lead to reductions in screen use, as well as improvements in physical activity, reductions in sedentary time, and better sleep [11–13].

Meta-analyses show interventions targeting reductions in children’s screen time, either alone or as part of a multi-behavioral intervention, are effective in reducing children’s body mass index (BMI) and decreasing children’s screen time [11, 14]. Despite evidence on the effectiveness of interventions to reduce children’s screen time [11, 13, 14], it remains unclear what study characteristics and behavior change techniques are the most critical to include in the design of screen time interventions. Behavior change techniques are the components or elements of an intervention, such as self-monitoring, social support, and signing behavioral contracts, that may serve as potential mechanisms underlying the effectiveness of behavioral interventions [15]. Although prior reviews provided initial evidence of the ability to intervene upon and reduce children’s and adolescents’ screen time, these reviews did not explore the potential mechanisms underlying the behavior changes documented in these interventions [11, 13, 14]. Identification of the behavior change techniques that are associated with maximal intervention effectiveness can be used to streamline and optimize the delivery of interventions to reduce screen time. Resources, such as time and money, may often be limited when designing and implementing behavioral interventions. Thus, identifying the “active” components of behavioral interventions can be used to maximize intervention results while minimizing the use of limited resources. The purpose of this systematic review and meta-analysis is to identify the behavior change techniques and study characteristics associated with treatment effectiveness in behavioral interventions to reduce children’s (0–18 years) screen time.

## Methods

The review process is reported according to the PRISMA 2020 guidelines statement [16]. This review protocol was not registered with PROSPERO.

### Search strategy

A comprehensive literature search was conducted between January and February 2020. Four databases (Ebscohost, Web of Science, EMBASE, and Ovid Medline/PubMed) were searched from their earliest record of publication through articles published in January 2020. The Ebscohost meta-database was used to search articles indexed in the following databases: Academic Search Complete, CINAHL Complete, CINAHL Plus with Full Text, ERIC, Health and Psychosocial Instruments, Health Source: Nursing/Academic Edition, MEDLINE with Full Text, PsycARTICLES, Psychology and Behavioral Sciences Collection, PsycINFO, PsycTESTS, Social Sciences Full Text, and Social Work Abstracts. The search strategy used a mixture of keywords, Boolean operators, and expanded vocabulary terms where appropriate for study design (intervention, trial, experiment, program), participants (child, preschool, adolescents, school, youth), and intervention target (television, computer, “media use,” “screen time,” “video game,” “recreational media,” and sedentary). To account for articles indexed after the initial search process, an updated search of articles published between February 2020 and July 2021 was performed in July 2021 using the original search strategy described above. The search strategy was developed by two authors (AJ and MB) and is provided in Additional file 1. Reference lists of systematic reviews and meta-analyses were reviewed to identify additional studies that were not captured in the initial search [14, 17–33]. The citations for identified articles were uploaded into an EndNote (version X9.2) library for reference management.

### Study inclusion criteria

Articles were eligible for review if they: 1) targeted children ≤ 18 years, 2) were a behavioral intervention that targeted a reduction in screen/sedentary time (i.e., television, video games, computer, etc.) or reported screen/sedentary time as an outcome, and 3) were published in an English, peer-reviewed journal. For this review, “screen/sedentary time” was defined as screen-based activities (i.e., television, video games, computer, etc.). The combination of screen/sedentary time was included as these behaviors are often conflated in the literature despite being distinct behavioral constructs. However, for the purposes of this review, articles targeting reductions in sitting time, without a focus on screens, were excluded. There were no geographic restrictions. In addition to randomized controlled trials (RCTs), quasi-experimental, single-group pre-post, and pilot studies were eligible for inclusion. Studies that took place in a lab or weight management clinic were excluded as the ability to generalize to free-living intervention studies is limited. The complete list of inclusion and exclusion criteria are provided in Additional file 2.

### Study identification

EndNote reference management software (version X9.2) was used to initially discard duplicate articles. Citations were then uploaded into the Covidence Systematic Review Management Program. Titles and abstracts were screened for relevancy by one reviewer (AJ), and those meeting initial inclusion criteria were forwarded to a full-text review by two independent reviewers (AJ and HP). Cases of disagreement were resolved by discussion between two reviewers (AJ and HP). Eligible articles were retained for qualitative and quantitative data extraction.

### Data extraction and quality assessment

Two authors independently (AJ and HP) extracted qualitative and quantitative data from the full text of eligible articles. Qualitative information regarding study-level characteristics (e.g., design, sample size, duration, participant characteristics, study location, self-identified pilot status), intervention characteristics (e.g., number of sessions, setting, intervention delivery, intervention recipient, screen device target), underlying theoretical framework (e.g., social cognitive theory [SCT]), and the behavior change techniques incorporated into the intervention (e.g., information, goal-setting, feedback, etc.) were extracted into a custom Microsoft Excel file (version 2012) developed for this review. The Abraham and Michie Taxonomy of Behavior Change Techniques [15] was used as the framework for extracting information on the individual behavior change techniques used in each article. As a second step, the individual behavior change techniques were grouped into behavior change clusters (i.e., Goals, Feedback, and Planning; Knowledge and Consequences; Behavioral Repetition and Practice; Social Comparison) based on the Abraham and Michie Hierarchical Behavior Change Technique Taxonomy [34]. Risk of bias in individual studies was examined by extracting information on whether a study was described as a randomized trial, whether the treatment allocation was concealed, and whether the participants or outcome assessors were blind to intervention group assignment. The NHLBI Study Quality Assessment Tool for Controlled Intervention Studies was used as a reference when extracting information on the risk of bias in individual studies [35]. Studies were considered “poor” quality if they did not meet these three criteria or if the study did not report on these criteria. Studies were considered “fair” quality if they met at least one or two of these criteria. Studies were considered “good” quality if they met all three of these criteria. Studies that are considered “poor” quality reflect a high risk of bias whereas studies that are considered “good” quality may have the least risk of bias. Qualitative data were coded into categorical variables in Stata (version 16) to be quantitatively analyzed. The codebook used for this review is provided in Additional file 3.

The I^2^ statistic was calculated as ${I}^{2}=\frac{Q-df}{Q}*100\%$, where Q is the chi-square test statistic and df is the corresponding degrees of freedom (Cochrane handbook). Generally accepted values for the interpretation of the I^2^ statistic were used, where 0–40% suggests non-impactful inconsistency, 30–60% may represent moderate heterogeneity, 50–90% may represent substantial heterogeneity, and 75–100% may represent considerable heterogeneity (Cochrane handbook). Visual inspection of funnel plots and Egger’s test for small-study effects were used to assess risk of small-study publication bias.

The reported screen time was extracted from each study. There was variability in how screen time was reported across studies. Some studies reported total daily screen time whereas other studies reported screen time during specific time periods (e.g., weekday versus weekend). Screen time could be reported as a cumulative total across all screen-based devices, or it could be reported as a “device-specific” estimate, such as the number of hours per day spent watching television or playing video games. Contextual information (i.e., time of day, device specific, weekday versus weekend) on how screen time was measured was extracted from each study. Means, standard deviations (SD), and sample sizes (N) at baseline and post-intervention were extracted for each treatment group. When 95% confidence intervals were reported, the SD was calculated using the following formula, $SD=\left(\sqrt{N}\right)*(\frac{Upper limit-Lower limit}{3.92})$. When an interquartile range (IQR) was reported, the SD was calculated using the following formula, $SD=\frac{Upper limit-Lower limit}{1.95}$. When a standard error (SE) was reported, the SD was calculated using the following formula, $SD=\left(\sqrt{N}\right)*SE$. Within- and between-group change scores were extracted, if presented. Discrete data were extracted as the event and total sample size, within each group, at baseline and post-intervention. Odds ratios and relative risks with the corresponding 95% confidence interval were extracted for discrete outcomes, where presented. When outcomes were reported at multiple timepoints, each measurement was recorded. Data were extracted for the overall sample and for each subgroup when presented.

### Data analysis

The standardized difference of the mean (SDM) was calculated for each observation. Observations were aggregated at the study level to create an average SDM for each study. The SDM for each study was used to calculate a summary effect for the random-effects meta-analysis using Comprehensive Meta-Analysis (version 3) software. All effects were coded to have positive effects representing a beneficial impact of the intervention on screen time (e.g., a decrease in screen time for the treatment group compared to the control group) and a negative effect indicated either 1) a greater reduction in screen time in the control group compared to the intervention group, or 2) a smaller amount of screen time at baseline compared to post-intervention. Meta-regressions were used to analyze the association between behavior change techniques, study-level characteristics, and intervention effectiveness. With a meta-regression, the techniques of simple regression, where outcome data is analyzed at a subject-level, are applied to the collection of identified studies, and outcome data is analyzed at the study-level [36]. Meta-regressions were used to explore whether the number and type of behavior change clusters (i.e., Goals, Feedback, and Planning; Knowledge and Consequences; Behavioral Repetition and Practice; Social Comparison), theoretical frameworks (e.g., SCT), intervention components (e.g., intervention delivery agent, intervention recipient) and study-level characteristics (e.g., sample size, duration, etc.) were associated with increased intervention effectiveness. For the present review, the dependent variable of interest was the intervention effectiveness – as measured with the SDM – and the covariates of interest were the behavior change techniques, theoretical frameworks, intervention components, and study-level characteristics previously identified. The meta-analysis package (meta) in Stata (version 16) was used to conduct the meta-regression analyses.

### Availability of data and materials

The datasets generated and analyzed during the current study are available from the corresponding author on reasonable request.

## Results

**Fig. 1:**
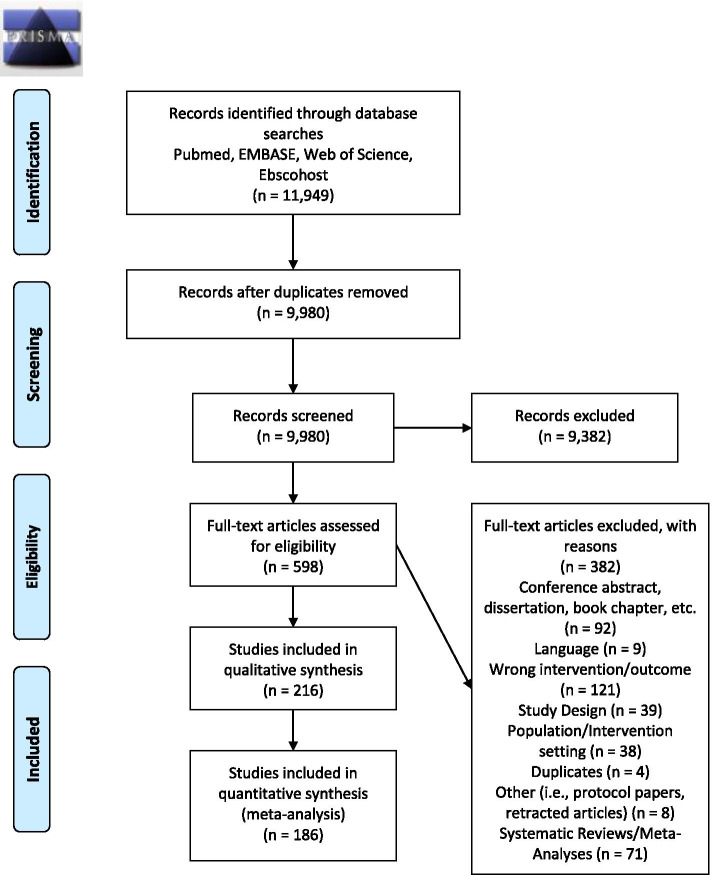
PRISMA 2009 Flow Diagram

**Fig. 2:**
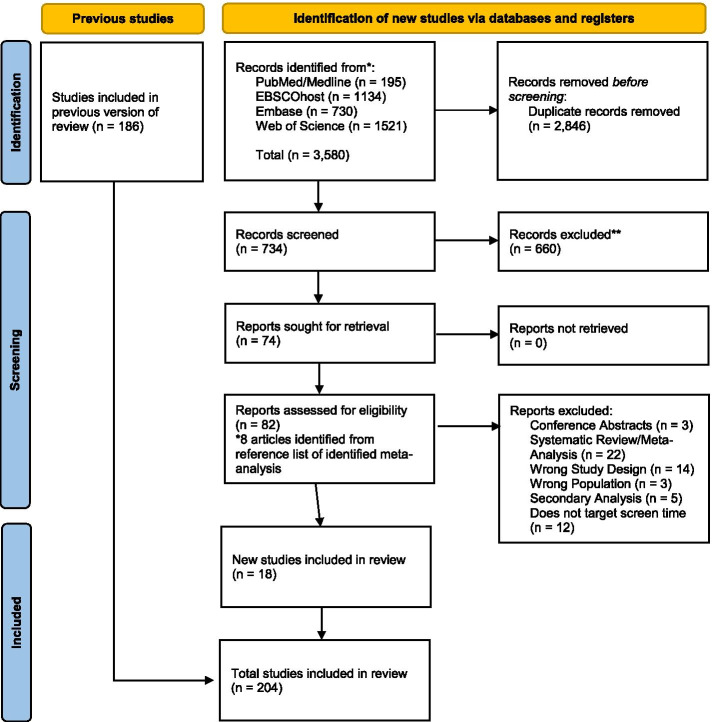
PRISMA 2020 Flow Diagram for Updated Systematic Reviews Which Included Searches of Databases and Registers Only

The original systematic search strategy identified 11,949 articles. After removing duplicates (*n* = 1,969), 9,980 articles were included in the initial title and abstract screen. Of the 9,980 articles screened for relevancy, 598 studies were retained for full-text eligibility screening, of which 287 studies were eligible for data extraction, and 216 articles were included in the systematic review. Of these 216 articles, 186 studies provided quantitative data that could be extracted and used in the meta-analysis. An additional 3,580 articles were identified in the updated search that was performed in July 2021. Of the 3,580 articles identified, 2,846 were duplicates, and 734 articles were included in the title and abstract screen. Of the 734 articles screened for relevancy, 82 were included in the full-text eligibility screening, and an additional 18 studies provided quantitative data for the meta-analysis. The meta-analysis included a final sample of 204 studies. An explanation of the criteria of exclusion for individual articles will be provided by the authors upon request. Study characteristics for the articles included in the meta-analysis are presented in Additional file 4. Figure 1 depicts the PRISMA flow chart of the initial screening process. Figure 2 depicts the PRISMA flow chart for the updated searches performed in July 2021.

### Study characteristics

Articles were published between 1998 and 2021. Nearly half of the studies took place in North America (k = 92, 45.1%) [37–126], with Europe (k = 49, 24.0%) [127–175], Oceania (k = 34, 16.7%) [176–209], Asia (k = 17, 8.3%) [210–226], South America (k = 11, 5.4%) [227–237], and Africa (k = 1, 0.5%) [238] also represented. There was considerable heterogeneity across the studies (I^2^ = 98.15%). Of the 204 articles included in the review, 147 studies (72%) self-identified as “randomized,” 22 studies (11%) provided information on allocation concealment, and 36 studies (18%) indicated that either the participant or outcome assessors research staff were blind to intervention group assignment. Of the included studies, 55 articles (27%) were “poor”, 136 (67%) were “fair” quality, and only 13 studies were considered “good” quality.

The average frequency represents the average across all studies included in the meta-analysis (k = 204). Across studies, there was variability in intervention duration (median = 24 weeks, IQR = 11 to 52 weeks). Interventions longer than 53 weeks (average frequency = 17%) were less common compared to interventions < 12 weeks (average frequency = 40%) and between 13–52 weeks (average frequency = 43%). Studies ranged considerably in sample size (*n* = 6 to *n* = 35,157; median = 317, IQR = 102 to 699) with 67 studies being self-identified pilot studies. Given the considerable range in the continuous sample size variable, a categorical sample size variable based on the interquartile range was created (*n* < 95, *n* = 96–312, *n* = 313–696, and *n* > 697).

The variation in study-level characteristics and intervention characteristics is presented by child age category in Table 1. Interventions delivered in schools were most common among adolescents 13 years and older (frequency = 86%). Interventions delivered by teachers were most reported in studies with children 6 years and older (frequency = 45%). Nearly all interventions (97%) used a subjective measure to quantify screen time. Interventions delivered to the parent only were most common in studies with children between 0 and 5 years old (frequency = 48%). The Social Comparison cluster was most common among studies with children 13 years and older (frequency = 93%). The Knowledge and Consequences cluster was most common among studies with children between the ages of 6 and 12 years (frequency = 97%). The Behavioral Repetition and Practice cluster was most common among studies with children 6 years and older (frequency = 84%). The Goals, Feedback, and Planning Cluster was most common among studies with children 13 years or older (frequency = 87%).

**Table 1:** Study-level and intervention characteristics by child age category

	**All Studies**	**0–5 years**	**6 + years**	**6–12 years**	$\le$**12 years**	**13 + years**	**0–18 years**
**k**	**204**	**46**	**31**	**80**	**24**	**15**	**7**
**Intervention Duration**							
< 16 weeks	40%	46%	48%	39%	38%	33%	14%
16–52 weeks	43%	37%	42%	45%	33%	60%	57%
> 53 weeks	17%	17%	10%	116%	29%	7%	29%
**Sample Size**							
< 95	25%	30%	19%	25%	25%	13%	43%
96–312	25%	30%	10%	25%	33%	40%	29%
313–696	25%	30%	32%	19%	29%	27%	14%
> 697	25%	10%	39%	31%	13%	20%	14%
**Self-Identified Pilot**	33%	43%	13%	29%	33%	40%	71%
**Setting**							
School/Daycare	59%	43%	74%	70%	29%	86%	14%
Home & mHealth	16%	26%	10%	11%	12%	14%	58%
Primary Care	10%	20%	6%	4%	17%	0%	14%
Community/Research Center	15%	11%	10%	15%	42%	0%	14%
**Study Design**							
Two-Group Randomized	68%	70%	77%	64%	79%	74%	29%
Two-Group, Non-Randomized	18%	17%	3%	24%	8%	13%	42%
Single-Group, Non-Randomized	14%	13%	20%	12%	13%	13%	29%
**Intervention Delivery**							
Teachers	32%	17%	45%	41%	17%	27%	14%
Research Staff	17%	13%	10%	24%	17%	13%	14%
Healthcare Professionals	21%	37%	6%	15%	33%	7%	43%
Other	30%	33%	39%	20%	33%	53%	29%
**Intervention Recipient**							
Child Only	54%	17%	84%	66%	29%	100%	29%
Parent Only	17%	48%	0%	5%	38%	0%	0%
Child and Parent	29%	35%	16%	29%	33%	0%	71%
**Screen Target**							
Targeted Other Obesogenic Behavior	37%	28%	52%	39%	25%	60%	13%
TV only	25%	39%	10%	24%	29%	7%	29%
TV & Other device	17%	11%	16%	23%	13%	13%	29%
Screen Time, General	21%	22%	22%	15%	33%	20%	29%
**Measurement Tool**		96%	100%	95%	100%	100%	86%
Self- or Parent-Report	97%	4%	0%	5%	0%	0%	14%
Objective Tool	3%						
**Social Comparison Cluster**	84%	80%	90%	83%	83%	93%	71%
**Knowledge and Consequences Cluster**	91%	83%	94%	97%	92%	93%	57%
**Behavioral Repetition/Practice Cluster**	73%	59%	84%	79%	67%	73%	57%
**Goals, Feedback, Planning Cluster**	78%	80%	81%	74%	83%	87%	86%
**Theoretical Frameworks**							
Social Cognitive Theory	41%	37%	48%	43%	29%	53%	29%
Social Ecological Model	15%	13%	16%	16%	21%	0%	14%
Multiple Theories	21%	24%	35%	16%	21%	13%	14%

The overall summary of random effects demonstrated a small, positive impact of screen time interventions compared to controls (SDM = 0.116, 95CI 0.08 to 0.15). The impact of study-level characteristics on intervention effectiveness is presented in Table 2. The largest treatment effects were observed in interventions delivered to children 12 years old or younger (k = 24, SDM = 0.209, 95CI 0.05 to 0.37). Shorter studies (< 12 weeks SDM = 0.151, 95CI 0.0 to 0.23 and 13–52 weeks SDM = 0.113, 95CI 0.07 to 0.16) were associated with larger intervention effects compared to studies > 53 weeks in duration (SDM = 0.061, 95CI -0.03 to 0.15). As sample size increased, there was a corresponding decrease in the magnitude of intervention effectiveness (*n* < 95, SDM = 0.298, 95CI 0.20 to 0.39 vs. *n* > 697, SDM = 0.054, 95CI 0.02 to 0.09). Interventions delivered by research staff (k = 35, SDM = 0.300, 95CI 0.16 to 0.44) and interventions delivered by other individuals, such as community members (k = 61, SDM = 0.149, 95CI 0.09 to 0.21), demonstrated a significant positive impact on children’s screen time.

**Table 2:** Estimate of intervention effectiveness by study-level characteristics

**Presence of Characteristic**	**k**	**% of studies**	**SMD**	**(95CI)**
**Overall Pooled Random Effect**	204	100%	0.116	**(0.08 0.15)**
**Child Age**				
0–5 years	46	23%	0.096	(-0.00 0.20)
6 + years	31	15%	0.200	**(0.16 0.28)**
6–12 years	80	39%	0.084	**(0.04 0.13)**
$\le$ 12 years	24	12%	0.209	**(0.05 0.37)**
13 + years	15	7%	0.103	**(0.02 0.18)**
0–18 years	7	3%	-0.047	(-0.59 0.49)
**Intervention Duration**				
< 12 weeks	67	33%	0.151	**(0.07 0.23)**
13–52 weeks	103	50%	0.113	**(0.07 0.16)**
> 53 weeks	34	17%	0.061	(-0.03 0.15)
**Sample Size**				
< 95	50	25%	0.298	**(0.20 0.39)**
96–312	52	25%	0.112	**(0.02 0.20)**
313–696	50	25%	0.063	(-0.01 0.14)
> 697	50	25%	0.054	**(0.02 0.09)**
**Setting**				
School/Daycare	121	59%	0.098	**(0.05 0.14)**
Home & mHealth	33	16%	0.152	**(0.07 0.23)**
Primary Care	19	9%	0.015	(-0.18 0.21)
Community/Research Center	31	15%	0.192	**(0.11 0.27)**
**Study Design**				
Two-Group Randomized	139	68%	0.129	**(0.09 0.17)**
Two-Group, Non-Randomized	36	18%	0.064	(-0.05 0.18)
Single-Group, Non-Randomized	29	14%	0.106	**(0.03 0.18)**
**Intervention Delivery**				
Teachers	65	32%	0.051	(-0.00 0.10)
Research Staff	35	17%	0.300	**(0.16 0.44)**
Healthcare Professionals	43	21%	0.051	(-0.05 0.15)
Other	61	30%	0.149	**(0.09 0.21)**
**Intervention Recipient**				
Child Only	111	54%	0.076	**(0.03 0.12)**
Parent Only	35	17%	0.222	**(0.09 0.35)**
Child and Parent	58	28%	0.147	**(0.07 0.23)**
**Screen Target**				
Targeted Other Obesogenic Behavior	76	37%	0.120	**(0.08 0.16)**
TV only	50	25%	0.093	**(0.01 0.18)**
TV & Other device	35	17%	0.124	**(0.04 0.21)**
Non-specific screen target	43	21%	0.089	(-0.02 0.20)
**Measurement Tool**				
Self- or Parent-Report	197	97%	0.113	**(0.07 0.15)**
Objective Tool	7	3%	0.235	(-0.02 0.49)

### Intervention characteristics

The estimate of intervention effectiveness by the presence or absence of a behavior change techniques is presented in Table 3. Of the 21 potential behavioral techniques, the 3 most frequently included were information on the behavior health link (k = 168), instruction (k = 145), and social support (k = 134). Behavioral contracts (k = 13), prompting cues (k = 18), and motivational interviewing (k = 22) were the least frequently included behavior change techniques (see Table 3). Studies did not use behavior change techniques in isolation. On average, interventions included 8.0 (range 0 to 18) different behavior change techniques, with some interventions including as many as 18 of the 21 techniques. Using the Hierarchical Behavior Change Technique Taxonomy [34], 4 clusters of co-occurring behavior strategies were identified: 1) Goals, Feedback, and Planning, 2) Social Comparison, 3) Knowledge and Consequences, and 4) Behavioral Repetition and Practice. To be included in a cluster, studies had to use at least one of the individual behavior change techniques. The Knowledge and Consequences cluster was the most common cluster (k = 186) whereas the Behavioral Repetition and Practice cluster was the least common cluster (k = 148). Including the Goals, Feedback, and Planning cluster was associated with the largest impact on intervention effectiveness (k = 160, SDM = 0.145, 95CI 0.11 to 0.18) compared to those studies that did not include the Goals, Feedback, and Planning cluster (k = 44, SDM = 0.001, 95CI -0.11 to 0.11, test of difference SDM = 0.154, 95CI 0.07 to 0.24). The inclusion of the Social Comparison, Behavioral Repetition and Practice, or the Knowledge and Consequences clusters were not associated with greater intervention effectiveness when compared to interventions that did not include these clusters, respectively (see Table 3).

**Table 3:** Estimate of intervention effectiveness by the presence or absence of behavior change clusters or techniques

	**Presence of Characteristic**				**Absence of Characteristic**			**Test of Difference**	
	**k**	**% of studies**	**SMD**	**(95CI)**	**k**	**SMD**	**(95CI)**	**SMD**	**(95CI)**
**Overall Pooled Random Effect**	204	100%	0.116	**(0.08 0.15)**					
**Motivational Interviewing (Package)**	22	11%	0.155	**(0.00 0.31)**	182	0.112	**(0.07 0.15)**	0.030	(-0.09 0.15)
**Social Comparison Cluster**	171	84%	0.119	**(0.08 0.16)**	33	0.098	**(0.00 0.19)**	0.021	(-0.08 0.12)
Social Comparison	51	25%	0.150	**(0.09 0.21)**	153	0.105	**(0.06 0.15)**	0.051	(-0.03 0.14)
Social Support	134	66%	0.112	**(0.07 0.15)**	70	0.123	**(0.04 0.20)**	0.001	(-0.08 0.08)
Modeling	99	49%	0.102	**(0.04 0.16)**	105	0.124	**(0.08 0.17)**	-0.030	(-0.11 0.05)
Encouragement	106	52%	0.125	**(0.07 0.18)**	98	0.107	**(0.05 0.16)**	0.021	(-0.05 0.10)
Role Model	76	37%	0.129	**(0.09 0.17)**	128	0.111	**(0.05 0.17)**	0.031	(-0.05 0.11)
**Knowledge and Consequences Cluster**	186	91%	0.116	**(0.08 0.15)**	18	0.138	(-0.06 0.34)	0.019	(-0.12 0.16)
Information on Behavior-Health Link	168	82%	0.111	**(0.07 0.15)**	36	0.175	**(0.03 0.32)**	-0.018	(-0.12 0.08)
Information on Consequences	68	33%	0.162	**(0.10 0.22)**	136	0.093	**(0.04 0.14)**	0.073	(-0.01 0.15)
Instruction	145	71%	0.120	**(0.08 0.16)**	59	0.105	**(0.03 0.18)**	0.019	(-0.06 0.10)
**Behavioral Repetition/Practice Cluster**	148	73%	0.117	**(0.07 0.16)**	56	0.112	**(0.04 0.18)**	0.005	(-0.08 0.09)
Prompts Cues	18	9%	0.147	**(0.01 0.28)**	186	0.113	**(0.07 0.15)**	0.035	(-0.10 0.17)
Prompts Practice	129	63%	0.109	**(0.06 0.16)**	75	0.127	**(0.07 0.19)**	-0.021	(-0.10 0.06)
Graded Tasks	28	14%	0.234	**(0.15 0.32)**	176	0.098	**(0.06 0.14)**	0.145	**(0.03 0.26)**
Contingent Rewards	57	30%	0.133	**(0.08 0.18)**	147	0.107	**(0.06 0.16)**	0.044	(-0.04 0.13)
**Goals, Feedback, Planning Cluster**	160	88%	0.145	**(0.11 0.18)**	44	0.001	(-0.11 0.11)	0.154	**(0.07 0.24)**
Goal Setting	97	48%	0.182	**(0.13 0.23)**	107	0.058	**(0.01 0.11)**	0.129	**(0.06 0.20)**
Goal Review	38	19%	0.222	**(0.14 0.30)**	166	0.092	**(0.05 0.13)**	0.131	**(0.04 0.23)**
Feedback	60	29%	0.137	**(0.08 0.19)**	144	0.107	**(0.06 0.16)**	0.040	(-0.04 0.12)
Self-Monitoring	84	41%	0.183	**(0.13 0.24)**	120	0.068	**(0.02 0.12)**	0.118	**(0.04 0.19)**
Behavioral Contract	13	6%	0.139	**(0.03 0.24)**	191	0.114	**(0.07 0.15)**	0.041	(-0.11 0.19)
Intention Formation	81	40%	0.154	**(0.11 0.19)**	123	0.089	**(0.03 0.15)**	0.083	**(0.01 0.16)**
Barrier Identification	80	39%	0.115	**(0.08 0.15)**	124	0.113	**(0.05 0.17)**	0.024	(-0.05 0.10)
Follow-Up Prompts	85	42%	0.175	**(0.12 0.23)**	119	0.074	**(0.03 0.12)**	0.103	**(0.03 0.18)**
**Theoretical Frameworks**									
Social Cognitive Theory	83	41%	0.139	**(0.10 0.18)**	121	0.101	**(0.04 0.16)**	0.053	(-0.02 0.13)
Social Ecological Model	30	15%	0.096	**(0.03 0.16)**	174	0.119	**(0.08 0.16)**	-0.013	(-0.12 0.09)
Multiple Theories	43	21%	0.131	**(0.06 0.20)**	161	0.113	**(0.07 0.16)**	0.022	(-0.07 0.11)

Follow-up analyses were conducted to examine how study-level characteristics of intervention duration, sample size, and intervention delivery agent modified the impact of including the Goals, Feedback, and Planning cluster on intervention effectiveness (see Table 4). As intervention duration increased from < 12 to > 53 weeks, the influence of having the Goals, Feedback, and Planning behavioral cluster dampened (SDM = 0.182, 95CI 0.12 to 0.25 versus SDM = 0.108, 95CI 0.05 to 0.17). The inclusion of the Goals, Feedback, and Planning cluster amplified the magnitude of effect for studies < 12 weeks (test of difference SDM = 0.200, 95CI 0.02 to 0.38) and > 53 weeks (test of difference SDM = 0.244, 95CI 0.04 to 0.45) in duration compared to studies that did not include this cluster.

**Table 4:** Impact of study-level and intervention characteristics and goals, feedback, and planning cluster on intervention effectiveness

**Study-Level Characteristics**	**Presence of Characteristic with Goals, Feedback, and Planning Cluster**			**Absence of Characteristic with Goals, Feedback, and Planning Cluster**			**Test of Difference**	
	**k**	**SDM**	**(95CI)**	**k**	**SDM**	**(95CI)**	**SDM**	**(95CI)**
**Duration**								
< = 12 weeks	53	0.182	**(0.12 0.25)**	14	0.000	(-0.28 0.28)	0.200	**(0.02 0.38)**
13–52 weeks	80	0.140	**(0.08 0.19)**	23	0.031	(-0.05 0.11)	0.103	(-0.00 0.21)
> 53 weeks	27	0.108	**(0.05 0.17)**	7	-0.140	(-0.49 0.21)	0.244	**(0.04 0.45)**
**Intervention Delivery**								
Teachers	43	0.087	**(0.05 0.13)**	22	-0.013	(-0.15 0.12)	0.104	(-0.00 0.21)
Research Staff	31	0.345	**(0.20 0.49)**	4	-0.005	(-0.19 0.18)	0.337	(-0.07 0.74)
Healthcare Professionals	39	0.106	**(0.04 0.17)**	4	-0.445	(-1.22 0.33)	0.491	**(0.21 0.77)**
Other	47	0.160	**(0.09 0.23)**	14	0.110	**(0.01 0.21)**	0.039	(-0.10 0.18)
**Sample Size**								
< 95	44	0.306	**(0.20 0.41)**	5	0.25	(-0.01 0.51)	0.049	(-0.25 0.35)
96–312	44	0.141	**(0.08 0.21)**	8	-0.047	(-0.57 0.47)	0.230	(-0.02 0.48)
313–696	35	0.12	**(0.05 0.19)**	15	-0.066	(-0.24 0.11)	0.184	**(0.03 0.34)**
> 697	35	0.062	**(0.02 0.11)**	15	0.035	(-0.04 0.11)	0.027	(-0.05 0.11)

Interventions delivered by research staff that included the Goals, Feedback, and Planning cluster were significantly associated with reductions in children’s screen time (k = 28, SDM = 0.341, 95CI 0.18 to 0.50). Interventions delivered by healthcare professionals that included the Goals, Feedback, and Planning cluster demonstrated a significant, positive impact on screen time compared to interventions delivered by healthcare professionals without the Goals, Feedback, and Planning cluster (test of difference SDM = 0.500, 95CI 0.21 to 0.79). Sample size attenuated the impact of the Goals, Feedback, and Planning cluster, with small studies (*n* < 95) driving the magnitude of effect (SDM = 0.306, 95CI 0.20 to 0.41) and tapering off as sample size increased (*n* > 697; SDM = 0.062, 95CI 0.02 to 0.11). Across studies of the same sample size, those that included the Goals, Feedback, and Planning cluster demonstrated larger intervention effects compared to studies that did not include this cluster. However, this effect was only significant when sample sizes were between 313–696 participants (test of difference SDM = 0.184, 95CI 0.03 to 0.34).

**Fig. 3:**
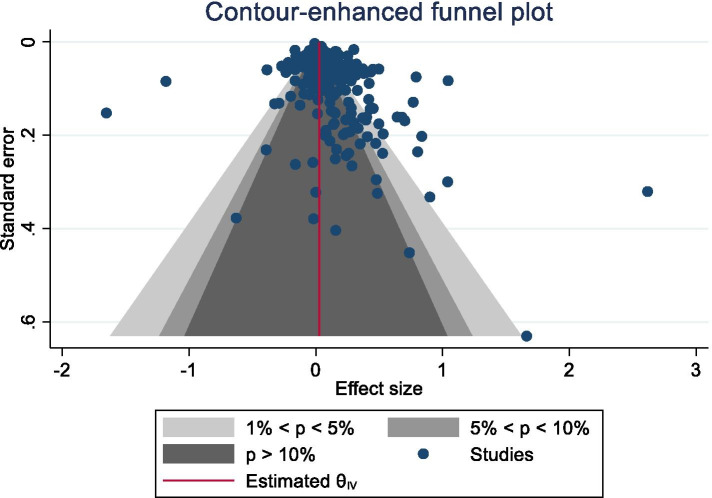
Contour-Enhanced Funnel Plot for Publication Bias

Visual inspection of the contour-enhanced funnel-plot suggested evidence of publication bias due to small-study effects (see Fig. 3). Egger’s test for small-study effects highlighted significant evidence of small-study publication bias (*p* < 0.001). The trim-and-fill analysis estimated 58 studies were missing due to publication bias, with the imputed studies reducing the overall SDM from 0.116 (95CI 0.08 to 0.15) to 0.007 (95CI -0.04 to 0.05).

## Discussion

The current meta-analysis found an overall small, positive effect of screen time interventions, compared to controls, for reducing children’s screen time. The magnitude and direction of effects in the current meta-analysis is consistent with prior meta-analyses of behavioral interventions to reduce children’s screen time [11, 13, 14, 239]. The current study uniquely contributes to the literature by including children from birth through 18 years and identifying which behavior change techniques and study-level characteristics are associated with intervention effectiveness. The findings that the presence of goal setting strategies along with study-level characteristics (i.e., sample size, duration, and intervention delivery agent) are associated with larger improvements in children’s screen time have important implications for the content and design of interventions.

To optimize intervention effectiveness and maximize resources, an understanding of the “active ingredients” in behavioral interventions to reduce children’s screen time is warranted. We found that study characteristics such as smaller sample sizes and shorter intervention durations were associated with larger effects. Previous meta-analyses have shown the magnitude of intervention effectiveness decreased as sample size increased and that interventions with shorter durations (< 7 months) are more effective at reducing children’s screen time compared to interventions with longer durations [11, 240]. Collectively these studies suggest delivering a behavioral intervention under “ideal” conditions may be a primary ingredient to obtaining statistically significant improvements in children’s screen time. Our findings are aligned with recent reviews which have suggested that as interventions progress from the pilot stage to a larger, well-powered trial, there is a corresponding drop in the magnitude of the intervention effects [241]. Features such as small sample sizes and short intervention durations, which are characteristic of pilot studies, may introduce bias into the interpretation of the effectiveness of the intervention [241]. Subsequently, the conclusions drawn from these pilot studies can impact decisions regarding the scalability and generalizability of the intervention.

Although intervention context and delivery are important, intervention *content* (i.e., specific behavior change techniques), can modify the intervention effectiveness. Behavioral interventions that included techniques such as goal setting, goal review, and self-monitoring had larger effects compared to interventions that did not include these techniques. These findings are consistent with a recent review that identified goal setting, positive reinforcement, and family social support as active ingredients in behavioral interventions targeting reductions in children’s sedentary behavior [240]. Some techniques, such as goal setting, may be effective in reducing both children’s sedentary time *and* screen time. Yet, it is important to note that not all sedentary time involves screens. Thus, different behavioral strategies may be required to differentially target these often conflated and overlapping behavioral constructs.

The inclusion of the Goals, Feedback, and Planning cluster was the driving behavioral strategy associated with enhanced intervention effectiveness. Yet, there was variability across interventions in how this cluster of behavior change techniques was delivered during the intervention. For example, Dennison et al. [53] had participants identify alternative activities to television viewing whereas studies by Gorin et al. [73] and Morgan et al. [195] used a combination of goal-setting and self-monitoring to reduce children’s screen time. Thus, it is unclear whether some individual behavior change techniques, such as goal review, may be more effective at reducing children’s screen time compared to other techniques, such as barrier identification. Further, it is unclear whether there is a linear association between the number of individual behavior change techniques incorporated in an intervention and intervention effectiveness. Over half the studies (88%) included the Goals, Feedback, and Planning cluster in combination with another behavior change cluster indicating the use of this behavior change technique did not occur in isolation. Goal setting may catalyze behavior change by serving as the “initial step” in the behavior change process. Goal setting may represent the starting point from which actionable steps can be taken to reach the destination of behavior change.

There are a variety of hypothesized mechanisms relating goal setting to behavior change. Some authors [242] suggest motivation is the link between goal setting and behavior. Without motivation, individuals will not set goals and make plans to modify their behavior. Other authors [243] suggest goal setting should be paired with a complementary behavior change technique, such as feedback, to create a feed-forward mechanism that synergistically contributes to behavior change. A recent meta-analysis suggested that goal setting, in combination with feedback, behavioral repetition and practice, instruction, or modeling did not increase intervention effectiveness compared to interventions that included goal setting as the sole behavior change strategy [243]. However, the combination of goal setting with self-monitoring was found to strengthen the effect of the intervention [243]. Although goal setting is intertwined with other behavioral strategies, such as feedback and modeling, not all pairings of complementary behavior change techniques are equal. The cyclical nature of goal setting and self-monitoring may reflect the dynamic and ongoing process of behavior change that is not reflected when goal setting is paired with other behavior change techniques. Future studies should attempt to disentangle the additive effects of complementary behavior change techniques in relation to their impact on the effectiveness of behavioral interventions.

The current study examined the interaction between intervention content and a single domain of intervention context (i.e., duration, sample size, intervention delivery agent). Among studies that included the Goals, Feedback, and Planning cluster, there was a significant decrease in the magnitude of treatment effects as intervention duration increased from < 12 to > 53 weeks. Of the studies that included the Goals, Feedback, and Planning cluster, interventions delivered by research staff were associated with the largest treatment effects. Further, there was a descending magnitude of treatment effects as sample size increased for those studies that included the Goals, Feedback, and Planning cluster. In the absence of the Goals, Feedback, and Planning cluster, the impact of intervention duration, intervention delivery, and sample size was non-significant.

Behavioral interventions are complex, and the decision of which behavioral strategies to use may depend on the factors such as who the intervention is being delivered to and what resources are available to deliver the intervention. As researchers develop interventions, strategies that may be feasible to deliver to small samples may be impractical to deliver to larger samples. For example, strategies such as motivational interviewing and barrier identification may require intense, one-on-one contact between intervention personnel and participants. Further, strategies such as setting goals, providing graded tasks, or establishing a behavioral contract are often tailored to an individual’s needs. Screen use, including the timing, the amount, and the type of device used is highly variable within an individual. Thus, the use of highly tailored behavioral strategies may lead to larger improvements in screen time at the individual level. However, in studies delivered in group settings or with larger samples, the behavioral strategies may be adapted. Strategies such as social support and role modeling may be incorporated into larger trials due to the qualities of accountability and comparison that are inherently linked with these strategies. The decision to modify the behavioral strategies or the “ingredients” of the intervention may result in a corresponding change in the effectiveness of the intervention. As interventions are scaled, researchers should explore how a change in an intervention’s behavioral strategies or intervention “ingredients” impact the effectiveness of the intervention.

The current study is among the first to identify the behavioral strategies and study-level characteristics associated with the effectiveness of interventions to reduce children’s screen time. This meta-analysis was guided by a widely used taxonomy of behavior change techniques [15, 34], included 204 studies, and included screen time from multiple devices as an outcome. However, the current meta-analysis is not without its limitations. We acknowledge that this review protocol was not registered a priori with the PROSPERO systematic review trial registry. However, the PROSPERO database was searched to identify whether similar reviews were registered to avoid unintended duplication.

There were insufficient sample sizes to examine subgroup outcomes stratified by measurement tool. Almost all interventions (97%) measured screen time via self- or parent-report. These findings are consistent with a recent review that reported that no articles used an objective, device-based tool to measure screen time in children 6 years old or younger [244]. Interventions that objectively measured screen time were associated with larger, albeit non-significant effects, compared to interventions that measured screen time via self- or parent-report. The difference in the magnitude of effectiveness between measurement tools may be due to self-report or recall bias. New technology, such as passive mobile sensing, may improve the ability to objectively monitor screen time [245, 246]. As objective measures of screen time are developed and disseminated to researchers, future studies may explore how the precision of the measurement tool used to quantify screen time is associated with intervention effectiveness.

The current meta-analysis highlights the impact intervention delivery agents and intervention duration have on an intervention’s impact when compared to interventions delivered by individuals with less expertise or interventions delivered over longer durations. Our results suggest that larger treatment effects are observed in smaller studies whose intervention delivery, duration, and setting are often tightly controlled. These smaller studies are often “pilot studies,” a primary function of which is to aid decisions regarding the scaling up of an intervention [241]. During the transition from small, “pilot” interventions to larger efficacy trials, there may be changes in intervention delivery that are associated with a corresponding drop in the magnitude of intervention effectiveness [241]. These seemingly small decisions can introduce bias when interpreting the effectiveness of the larger intervention. Decisions to modify the components of a pilot study when scaled often occur due to funding constraints or logistical requirements for collecting and delivering an intervention to a larger sample [247]. Yet before a decision is made, researchers may want to carefully consider the consequences of tinkering an intervention that demonstrated promise in the pilot phase. Researchers can begin by thinking with “the end in mind” and design pilot study protocols that will be mirrored and implemented in the larger trial. If we intend for the results obtained in small studies to inform and generalize to larger scaled trials, then we need to be designing such studies with an eye toward scalability.

As the technological landscape continues to rapidly evolve, the way children and adolescents interact with technology will continue to change. Rather than simply designing interventions to reduce “screen time” which can apply to any electronic, screen-based activity, future researchers may consider designing targeted interventions that are device-specific. Further, increased access and use of screens can also modify how interventions to reduce screen time are delivered. Researchers are no longer constrained to in-person settings but can use mHealth or mobile platforms to deliver an intervention. Thus, the setting or the medium through which an intervention is delivered may modify the effectiveness of the intervention. Although mHealth interventions may increase the accessibility of a behavioral intervention, the use an electronic device to reduce screen time may be negated by the need to interact with the device in order to receive the intervention material. Future researchers may consider exploring whether using technology to deliver an intervention targeted at reducing screen time negates or amplifies the effectiveness of the intervention.

Finally, the current study found a small, positive effect of interventions to reduce children’s screen time, yet it is unknown whether the effect is clinically meaningful. International recommendations suggest children and adolescents (5–17 years old) engage in $\le$ 2 h per day of screen time (5). However, these guidelines were based on perceived population-level associations between screen time and other negative obesogenic behaviors, such as physical inactivity, and rates of overweight and obesity. Clinical meaningfulness is often based on the public health impact of an intervention or the average improvement in a behavior for a large group of individuals. Although small improvements at the population-level may be beneficial, there is considerable within-person variability in screen time, including what devices are used, when they are most frequently used, and how much screen time is being accumulated. Identification for whom and at what times screen time is most problematic may result in larger improvements in screen time at the person level. As science continues to move forward, researchers should consider whether they are designing public health interventions or person-level interventions.

Overall, behavioral interventions to reduce children’s screen time appear effective. As interventions are scaled, it is important to determine whether the catalyzing effect of specific behavioral strategies on reducing children’s screen time remains significant. Smaller studies have the advantage of stronger internal validity in terms of sample selection or delivery agent, may have potentially larger effect sizes. Factors that are highly controlled and that contribute to the internal validity in smaller studies are the same factors that reduce the ecological validity of these studies. The decision to tinker with these components during the process of scaling smaller studies make it untenable to assume that the effects documented in small studies will generalize to larger trials. If the purpose of a pilot study is to inform the decision to translate the intervention to larger trials, researchers should be designing pilot interventions with an emphasis on scalability and generalizability. Neglecting or minimizing the importance of scalability in the pilot design phase will continue to result in a literature base that shows that for a small number of people, under specific conditions (i.e., intervention setting, duration, delivery agent), with certain behavioral strategies, interventions *can* be effective. However, the public health implications of such a narrow purview are likely to be minimal. If our goal as public health researchers is to improve the health of the population, we need to expand and generalize our thinking during the early stages of intervention development to effectively utilize our resources and improve health outcomes for the maximum number of individuals.

## Supplementary Information

**Additional file 1.** Search Strategy.

**Additional file 2.** Inclusion Criteria and Exclusion Criteria.

**Additional file 3.** Codebook for Meta-Regression Analyses.

**Additional file 4.** Table of Included Studies.

**Additional file 5.** Summary Forest Plots.

## Data Availability

The datasets generated and analyzed during the current study are available from the corresponding author on reasonable request.
