# Supplementary material for: Identifying effective intervention strategies to reduce children’s screen time: a systematic review and meta-analysis
Source: Int J Behav Nutr Phys Act. 2021 Sep 16;18:126. doi: 10.1186/s12966-021-01189-6 (PMC8447784; doi:10.1186/s12966-021-01189-6)
Supplement: Supplementary file 1 — Additional file 1. Search Strategy. [file 12966_2021_1189_MOESM1_ESM.docx]

**Additional File 1**

**Ebscohost Search Strategy**

| **Study Design** | **Participants** | **Intervention Target** | **Reduction Term** | **Field** |
| --- | --- | --- | --- | --- |
| Trial  ***OR***  Program  ***OR***  Intervention  ***OR***  Experiment | Child*  ***OR***  Preschool*  ***OR***  Adoles*  ***OR***  School*  ***OR***  Youth | Television  ***OR***  Computer*  ***OR***  “Media Use”  ***OR***  “Screen Time”  ***OR***  “Video Game*”  ***OR***  “Recreational Media”  ***OR***  Sedentary | Reduc*  ***OR***  Limit | Title/Abstract |
| Example Search:  (trial [title/abstract] and child* [title/abstract] and television [title/abstract] and reduc* [title/abstract]) | | | | |

**Web of Science Search Strategy**

| **Study Design** | **Participants** | **Intervention Target** | **Reduction Term** | **Field** |
| --- | --- | --- | --- | --- |
| Trial  ***OR***  Program  ***OR***  Intervention  ***OR***  Experiment | Child*  ***OR***  Preschool*  ***OR***  Adoles*  ***OR***  School*  ***OR***  Youth | Television  ***OR***  Computer*  ***OR***  “Media Use”  ***OR***  “Screen Time”  ***OR***  “Video Game*”  ***OR***  “Recreational Media”  ***OR***  Sedentary | Reduc*  ***OR***  Limit | Title/Abstract |
| Example Search:  (program [title/abstract] and preschool* [title/abstract] and computer* [title/abstract] and limit* [title/abstract]) | | | | |

**EMBASE Search Strategy**

| **Study Design** | **Participants** | **Intervention Target** | **Reduction Term** | **Field** |
| --- | --- | --- | --- | --- |
| Trial  ***OR***  Program  ***OR***  Intervention  ***OR***  Experiment | Child*  ***OR***  Preschool*  ***OR***  Adoles*  ***OR***  School*  ***OR***  Youth | Television  ***OR***  Computer*  ***OR***  “Media Use”  ***OR***  “Screen Time”  ***OR***  “Video Game*”  ***OR***  “Recreational Media”  ***OR***  Sedentary | Reduc*  ***OR***  Limit | Title/Abstract |
| **Example Search**:  (intervention [title/abstract] and adoles* [title/abstract] and “media use” [title/abstract] and reduc* [title/abstract]) | | | | |

**OVID Medline/Pubmed Search Strategy**

| **Study Design** | **Participants** | **Reduction Term** | **Field** | **Intervention Target** | **Field** |
| --- | --- | --- | --- | --- | --- |
| Trial  ***OR***  Program  ***OR*** Intervention  ***OR***  Experiment | Child*  ***OR***  Preschool*  ***OR***  Adoles*  ***OR***  School*  ***OR***  Youth | Reduc*  ***OR***  Limit | Title/Abstract | Television  ***OR***  Computer*  ***OR***  “Media Use” ***OR***  “Screen Time”  ***OR***  “Video Game*”  ***OR*** “Recreational Media”  ***OR***  Sedentary | MeSH terms  MeSH terms  title/abstract  MeSH terms  MeSH terms  title/abstract  MeSH terms |
| **Example Search**:  (experiment [title/abstract] and school* [title/abstract] and limit [title/abstract] and “screen time” [MeSH terms] | | | | | |
