# Supplementary material for: Identifying effective intervention strategies to reduce children’s screen time: a systematic review and meta-analysis
Source: Int J Behav Nutr Phys Act. 2021 Sep 16;18:126. doi: 10.1186/s12966-021-01189-6 (PMC8447784; doi:10.1186/s12966-021-01189-6)
Supplement: Supplementary file 2 — Additional file 2. Inclusion Criteria and Exclusion Criteria. [file 12966_2021_1189_MOESM2_ESM.docx]

**Additional File 2**

**Inclusion Criteria**

- Behavioral intervention that targets a reduction in screen/sedentary time (i.e., television, video games, computer, phone, etc.) or reports screen/sedentary time (i.e., television, video games, computer, phone, etc.) as an outcome
- Targets children aged 0-18 years old (can include family-based interventions and interventions that target parents of infants/preschoolers)
- Published in a peer-reviewed, English-language journal

**Exclusion Criteria**

- Books, conference abstracts, pre-prints, dissertations, or master’s thesis
- Commentaries, clinical advice, or guideline papers
- Observational studies, secondary data analysis of surveillance data, non-experimental surveillance data
- Scale, tool, test, or device development
- Protocol paper, methods papers, or protocol/method validation papers
- Clinically disordered populations, including clinical diagnosis (e.g., cancer), psychological disorders (e.g., depression, mood disorders, eating disorders), and developmental disorders (e.g., Intellectual Developmental Disorder)
- Studies on non-human specimens (e.g., animals, viruses/bacteria, computer algorithms, mathematical simulation models)
- Intervention targeted adults ($>$18 years), who were not parents of or did not care for, children/adolescents (0-18 years)
- Intervention did not target a reduction in screen/sedentary time **or** did not report screen time/sedentary time as outcome
- Intervention targeted a reduction in sitting or seated time
