# Supplementary material for: Identifying effective intervention strategies to reduce children’s screen time: a systematic review and meta-analysis
Source: Int J Behav Nutr Phys Act. 2021 Sep 16;18:126. doi: 10.1186/s12966-021-01189-6 (PMC8447784; doi:10.1186/s12966-021-01189-6)
Supplement: Supplementary file 3 — Additional file 3. Codebook for Meta-Regression Analyses. [file 12966_2021_1189_MOESM3_ESM.docx]

**Additional File 3**

**Codebook for Meta-Regression Analyses**

**Study Design -** *design*

1 = Cluster RCT

2 = RCT

3 = Quasi-experimental

4 = Single-Group Pre-Post

*recode design (1/2=1) (3=2) (4=3)*

1 = Two-group, randomized

2 = Two-group, non-randomized designs

3 = Single Group Pre-Post

**Intervention Duration –** *duration_cat*

0 = $\leq$12 weeks

1 = 13 to 52 weeks

2 = $\geq$ 53 weeks

**Intervention Sessions –** *intervention_sessions*

*Report the max number of possible intervention sessions

**Intervention Session Duration** – *session_duration*

*Report the max length of the intervention session (in minutes)

**Intervention Dose** – *intervention_dose*

*Calculated as the number of intervention sessions multiplied by the session duration

**Study Setting** – *setting*

1 = School (includes after school/out of school programs)

2 = Home

3 = Preschool/daycare/nurseries (includes children’s centers, head start centers, surestart centers, playgroups)

4 = Community

5 = Primary Care Offices/Health Centers (include WIC, EFNEP, Community Health Centers)

6 = mHealth

7 = Research Center

8 = Any location with an mHealth component

9 = Home and a secondary location

*recode setting (3=1) (6=2) (8=2) (9=2) (7=4) (5=3)*

1 = School, preschool, daycare, nurseries

2 = Home & mHealth

3 = Primary care

4 = Community and research centers

**Socio-Ecological Framework Components**

0 = No

1 = Yes

*IndividualLevelknowledgeatt* - targets the individual's (i.e., child's) behaviors and attitudes.

*InterpersonalLevelfamiliesf* - targets the child's interpersonal relationships (i.e., family, social networks)

*OrganizationalLevelorganizati* - targets structural changes to the organization (i.e., changes to the school/daycare environment such as modifying PE classes, providing increased access to healthy diet/PA opportunities)

*CommunityLevelrelationshipsb* - targets changes to the community and incorporates community involvement (i.e., community events, changes to PA opportunities within the community)

*PublicPolicyLevelstateloca* - policy level changes

**Theoretical Framework**

0 = No

1 = Yes

*SCT* – Social Cognitive Theory

*SEM* – Social-Ecological Model

*SDT* – Self-Determination Theory

*FST* – Family Systems Theory

*Other_Theory* – Any theory but SCT

*Replace Other_Theory = 1 if SCT==0 & SEM==1*

*Replace Other_Theory = 1 if SCT==0 & SDT==1*

*Replace Other_Theory = 1 if SCT==0 & FST==1*

*Multiple_Theories* – Study uses more than 1 theoretical framework

*Replace Multiple_Theories = 1 if SCT==1 & Other_Theory==1*

**Intervention Target Behavior** – *intervention_target*

1 = Screen time

2 = Sedentary time

3 = Other (e.g., physical activity, diet, sleep), screen and sedentary time

**Co-Intervention**  - *IsthisaCoIntervention*

0 = No

1 = Yes

**Outcome Behavior** – *outcome_behavior*

0 = Did not report screen time

1 = Sedentary time

2 = Screen time

**Outcome Measurement (i.e., what tool did they use?)** – *outcome_measurement*

0 = Did not report screen time

1 = HBSC survey

2 = Questionnaire developed for study

3 = PA recall

4 = NHANES survey

5 = Florindo 2006 survey

6 = ASAQ

7 = INTA

8 = YRBS

9 = VCAMS

10 = Health Promotion Survey Northern Ireland

11 = Maine Youth Health Survey

12 = Promoting Healthy Lifestyles Survey

13 = Accelerometer

14 = NYLS

15 = FEAHQ

16 = CHIS

17 = Children's Leisure Activities Study Survey

18 = SPAN

19 = Todd and Currie Sedentary Behavior Survey

20 = EPAQ

21 = School-based Nutrition Monitoring Questionnaire

22 = TV time monitor

23 = Flemish validated questionnaire

24 = Fernandes 2012 survey

25 = Robinson 1999 survey

26 = Gentile 2002 survey

27 = Downing and Hinkley surveys

28 = CPAQ

29 = Time Use Diary

30 = Primary Caregiver Questionnaire (PCQ)

31 = HealthKick Questionnaire

32 = SAPAC

33 = Dennison 2004 survey

34 = Child Sedentary Behavior Questionnaire CSBQ

35 = KiGGS

36 = Borzekowski survey

37 = Neumark-Sztainer survey

38 = Healthy Habits Survey

39 = Healthy Kids Checklist

40 = Godin Leisture Time Activity Questionnaire

41 = Baranowski 2008 survey

42 = Slemenda survey

43 = Salmon 2005 survey

44 = Parent Survey and Time Use Diary

45 = Flood 2005 survey

46 = Modifiable Activity Questionnaire

47 = Pre-PAQ

48 = AQUAA

49 = Sedentary Behavior Scale

50 = Oken 2007 survey

51 = Screen time log

52 = Healthy Habits Survey

53 = LATE Survey

54 = Environmental Audit

55 = Sedentary Behavior Log

56 = Interview

*recode outcome_measurement (1/12 = 1) (13 = 2) (14/21 = 1) (22 = 3) (23/53 = 1) (54 = 4) (55/56 = 1), gen(measurement_tool)*

1 = Self- or Parent-Report

2 = Accelerometer

3 = TV Time Monitor

4 = Environmental Audit

*recode measurement_tool (3/4=2)*

1 = Self- or Parent-Report

2 = Objective Measurement Tool

**Outcome Report (i.e., who reported the outcome)** – *outcome_report*

1 = Child

2 = Parent

3 = Objective Measure (e.g., accelerometer, tv time monitor)

4 = Parent/Child both report

**Intervention Recipient** – *intervention_recipient*

1 = Child only

2 = Parent only

3 = Child and parent

*Only 1 study delivered the intervention to daycare staff, was added into category 3

**Intervention Delivery** – *intervention_delivery*

1 = Teachers (includes daycare teachers, daycare staff, trained childhood educators, afterschool program staff, school wellness coordinators)

2 = Research staff (includes "academic researchers") - include college students here

3 = Healthcare professionals (includes doctors, nurses, nurse practitioners, psychologists, MI counselors, counselors, mental health specialists, behavioral coaches, sports medicine specialists, dieticians, nutritionists, WIC counselors, child development specialists, nutrition educators, health educators/consultants, case manager)

4 = mHealth

5 = Peers (includes peer educators)

6 = Delivered by people across multiple domains (i.e., teachers+healthcare professionals, research staff+healthcare professionals, teachers+healthcare professionals+research staff)

7 = Trained facilitators, program leaders, professionals

9 = Government program

10 = Community members (includes community leaders, community health promoters, volunteers)

*recode intervention_delivery (5/10=4)*

1 = Teachers

2 = Research staff

3 = Healthcare professionals

4 = Other

**Sample Size** – *samplesize_cat*

0 = $\leq$ 95

1 = $\geq$ 96 $\leq$ 312

2 = $\geq$ 313 $\leq$ 696

3 = $\geq$ 697 $\leq$ 4333

Mean sample size = 822.2527, SD = 2747.812

Median sample size = 311.5

<25th percentile = sample size <95

26-50th percentile = 96-312

51-75th percentile = 313-696

76-95th percentile = 697-4333

*Chose to exclude the largest two sample size studies (N = 10,204 & N = 35, 157)

**Age**

0 = No

1 = Yes

*age0_5* – children ages 0-5 years included in the study

*age6_12* – children ages 6-12 years included in the study

*age13up* – children ages 13-18 years included in the study

*age_min* – lowest child’s age included in the study

*age_max* – max child’s age included in the study

*age_average* – average age of child included in the study

**Self-Identified Pilot Study (i.e., identified as a pilot, feasibility, exploratory, evidentiary, or proof-of-concept study)** – *SelfIdentifiedPilot*

0 = No

1 = Yes

**Country (i.e., report the country in which the study was performed)**

**Targets High Risk Population (e.g., low-income, minority, overweight/obese)** – *highrisk*

0 = No

1 = Yes

**Randomized Study** – *Randomization*

0 = No/Did not report/Not applicable

1 = Yes

**Allocation Concealment** – *AllocationConcealment*

0 = No/Did not report/Not applicable

1 = Yes

**Participant or Interviewer Blinding** – *Blinding*

0 = No/Did not report/Not applicable

1 = Yes

**Behavior Change Techniques – Based on the Abraham and Michie (2008) Taxonomy of Behavior Change Techniques**

0 = No

1 = Yes

*info_behaviorhealth*

*info_consequences*

*intention_formation*

*barrier_identification*

*encouragement*

*gradedtasks*

*instruction*

*modeling*

*goal_setting*

*goal_review*

*selfmonitoring*

*feedback*

*contingent_rewards*

*prompts_cues*

*behavioral_contract*

*prompt_practice*

*followup_prompts*

*social_comparison*

*social_support*

*role_model*

*motivational_interviewing*

*We need to collapse/condense these behavioral techniques into smaller categories.

**Education - info_behaviorhealth info_consequences instruction modeling

**Goal Setting, Self-Monitoring, and Goal Review - intentionformation barrier_identification goal_setting goal_review feedback selfmonitoring encouragement gradedtasks

**Motivational Interviewing - motivational_interviewing

**Social Support and Social Comparison - social_comparison social_support role_model

**Accountability/Prompting/Communication - prompt_practice prompts_cues followup_prompts behavioral_contract contingent_rewards

**Screen Target (i.e., what screens/devices were targeted during the intervention)** - *screentarget*

1 = TV only (tv/dvd/videos)

2 = TV & Computer

3 = TV & Phone

4 = TV & Videogames

5 = TV, Computer, Videogames

6 = TV, Computer, Phone

7 = Phone only

8 = TV, Computer, Videogames, Phone

9 = TV, Computer, Portable Electronic Device

*recode screentarget (1 = 1) (2 = 2) (4 = 3) (3 = 4) (5/9 = 4), gen(screen_targetr)*

0 = Did not target screen time

1 = TV only

2 = TV and another device

3 = Screen Time, General
