# Supplementary material for: Identifying effective intervention strategies to reduce children’s screen time: a systematic review and meta-analysis
Source: Int J Behav Nutr Phys Act. 2021 Sep 16;18:126. doi: 10.1186/s12966-021-01189-6 (PMC8447784; doi:10.1186/s12966-021-01189-6)
Supplement: Supplementary file 4 — Additional file 4. Table of Included Studies. [file 12966_2021_1189_MOESM4_ESM.docx]

| **Author** | **Country** | **Sample Size** | **Intervention Duration (Weeks)** | **Screen Target** | **Outcome** | **Number of Strategies** | **Age Min.** | **Age Max.** | **Randomize** | **Allocation Concealment** | **Blinding** | **Risk of Bias** |
| --- | --- | --- | --- | --- | --- | --- | --- | --- | --- | --- | --- | --- |
| Adams (2018) | US | 291 | 52 | tv, computer | screen time | 6 | 0 | 2 | 1 | 0 | 0 | 1 |
| Aitassalo (2019) | Finland | 1550 | 4 |  | screen time | 10 | 13 | 14 | 1 | 0 | 0 | 1 |
| Amini (2016) | Iran | 326 | 18 |  |  | 4 | 10 | 12 | 1 | 0 | 0 | 1 |
| Anand (2007) | Canada | 174 | 24 | tv, videogames, computer | screen time | 7 | 5 | 18 | 1 | 0 | 0 | 1 |
| Andrade (2015) | Ecuador | 1370 | 112 | tv | screen time | 7 | 12 | 15 | 1 | 0 | 0 | 1 |
| Atalla (2018) | Brazil | 3592 | 28 | tv, videogames | tv/dvd/vidoes | 9 | 6 | 17 | 0 | 0 | 0 | 0 |
| Babic (2016) | Australia | 322 | 24 |  | screen time | 14 | 13 | 14 | 1 | 0 | 0 | 1 |
| Bacardi-Gascon (2012) | Mexico | 532 | 24 | tv | tv/dvd/vidoes | 4 | 7 | 9 | 1 | 0 | 0 | 1 |
| Backlund (2011) | Sweden | 105 | 104 |  | screen time | 18 | 8 | 12 | 1 | 0 | 0 | 1 |
| Bagherniya (2018) | Iran | 172 | 30 |  | sedentary time | 17 | 12 | 16 | 1 | 0 | 0 | 1 |
| Bandeira (2020) | Brazil | 1085 | 16 |  | screen time | 7 | 11 | 17 | 1 | 0 | 0 | 1 |
| Baumgartner (2020) | Germany | 486 | 156 |  | screen time | 3 | 8 | 9 | 0 | 0 | 0 | 0 |
| Bhave (2016) | India | 1056 | 260 |  | tv/dvd/vidoes | 7 | 7 | 10 | 0 | 0 | 0 | 0 |
| Bickham (2018) | US | 479 | 6 |  |  | 13 | 11 | 13 | 0 | 0 | 0 | 0 |
| Birken (2012) | Canada | 160 | 52 |  | screen time | 12 | 3 | 3 | 1 | 1 | 1 | 3 |
| Bjelland (2011) | Norway | 1580 | 32 |  | tv/dvd/vidoes | 9 | 11 | 13 | 1 | 0 | 0 | 1 |
| Bolton (2017) | Australia | 2964 | 156 |  | tv/dvd/vidoes | 13 | 5 | 18 | 0 | 0 | 0 | 0 |
| Breslin (2012) | Ireland | 416 | 12 |  | tv/dvd/vidoes | 9 | 8 | 9 | 0 | 0 | 0 | 0 |
| Brotman (2012a) | US | 99 | 24 |  |  | 0 | 3 | 5 | 1 | 0 | 0 | 1 |
| Brotman (2012b) | US | 496 | 24 |  |  | 0 | 3 | 5 | 1 | 0 | 0 | 1 |
| Brown (2018) | US | 23 | 11 | tv | screen time | 13 | 6 | 9 | 1 | 1 | 1 | 3 |
| Burke (1998) | Australia | 800 | 20 |  | tv/dvd/vidoes | 13 | 11 | 11 | 1 | 0 | 0 | 1 |
| Buscemi (2019) | US | 153 | 8 |  | tv/dvd/vidoes | 5 | 2 | 5 | 0 | 0 | 0 | 0 |
| Busch (2015) | Netherlands | 969 | 104 |  | tv/dvd/vidoes | 6 | 14 | 18 | 0 | 0 | 0 | 0 |
| Byrd-Bredbenner (2018) | US | 489 | 32 |  |  | 11 | 2 | 6 | 1 | 0 | 0 | 1 |
| Campbell (2013) | Australia | 542 | 60 | tv | tv/dvd/vidoes | 11 | 2 | 6 | 1 | 0 | 0 | 1 |
| Canavera (2009) | US | 122 | 12 | tv | tv/dvd/vidoes | 10 | 10 | 12 | 0 | 0 | 0 | 0 |
| Carson (2013) | Australia | 599 | 36 |  | sedentary time | 7 | 7 | 9 | 1 | 0 | 0 | 1 |
| Centis (2012) | Italy | 209 | 20 |  | tv/dvd/vidoes | 11 | 9 | 10 | 0 | 0 | 0 | 0 |
| Cespedes (2014) | US | 445 | 52 | tv. videos | tv/dvd/vidoes | 9 | 2 | 7 | 1 | 0 | 0 | 1 |
| Chen (2019) | US | 40 | 24 |  | screen time | 11 | 13 | 18 | 1 | 0 | 0 | 1 |
| Chin (2008) | Netherlands | 1108 | 32 | tv, computer |  | 14 | 12 | 13 | 1 | 0 | 0 | 1 |
| Cliff (2011) | Australia | 206 | 24 |  | screen time | 16 | 5 | 9 | 1 | 1 | 0 | 2 |
| Colin Ramirez (2010) | Mexico | 619 | 52 | tv, computer, videogames | tv/dvd/vidoes | 6 | 8 | 10 | 1 | 0 | 0 | 1 |
| Cong (2012) | US | 416 | 72 | tv, videogames | screen time | 14 | 5 | 9 | 0 | 0 | 0 | 0 |
| Contento (2007) | US | 278 | 8 |  | screen time | 15 | 11 | 13 | 0 | 0 | 0 | 0 |
| Contento (2010) | US | 1136 | 10 |  | screen time | 14 | 11 | 13 | 1 | 0 | 0 | 1 |
| Cronk (2011) | US | 54 | 6 | tv | tv/dvd/vidoes | 15 | 8 | 11 | 0 | 0 | 0 | 0 |
| Crouter (2015) | US | 43 | 10 |  | sedentary time | 13 | 9 | 10 | 1 | 0 | 1 | 2 |
| Cui (2012) | China | 682 | 4 |  | sedentary time | 7 | 12 | 13 | 1 | 0 | 1 | 2 |
| Cullen (2013) | US | 390 | 8 |  | computer | 8 | 12 | 17 | 1 | 0 | 0 | 1 |
| Davoli (2013) | Italy | 372 | 52 |  | screen time | 11 | 4 | 7 | 1 | 0 | 0 | 1 |
| De Coen (2012) | Belgium | 1589 | 104 |  | screen time | 7 | 3 | 6 | 1 | 0 | 0 | 1 |
| De Lima (2017) | Brazil | 47 | 12 |  |  | 3 | 10 | 19 | 1 | 0 | 0 | 1 |
| De Silva (2010) - Obesity | Australia | 18 | 208 |  |  | 5 | 0 | 5 | 0 | 0 | 0 | 0 |
| De Silva (2010) - reducing Obesity | Australia | 35157 | 208 | tv, DVD |  | 9 | 0 | 5 | 0 | 0 | 0 | 0 |
| Dennison (2004) | US | 176 | 7 | tv | tv/dvd/vidoes | 12 | 3 | 5 | 1 | 0 | 0 | 1 |
| Dewar (2013) | Australia | 357 | 52 |  | screen time | 5 | 12 | 13 | 1 | 0 | 0 | 1 |
| Dewar (2014) | Australia | 357 | 52 |  | tv/dvd/vidoes | 14 | 12 | 13 | 1 | 0 | 0 | 1 |
| Dickin (2014) | US | 210 | 84 |  | screen time | 8 | 3 | 11 | 0 | 0 | 0 | 0 |
| Downing (2018) | Australia | 57 | 6 |  | screen time | 11 | 2 | 4 | 1 | 1 | 0 | 2 |
| Duncan (2019) | New Zealand | 675 | 8 | tv, computer, videogames | tv/dvd/vidoes | 15 | 7 | 10 | 1 | 0 | 0 | 1 |
| Dunton (2009) | US | 695 | 8 | tv, videogames | screen time | 9 | 11 | 13 | 0 | 0 | 0 | 0 |
| Eagle (2013) | US | 4021 | 10 |  | tv/dvd/vidoes | 2 | 11 | 12 | 0 | 0 | 0 | 0 |
| Early (2019) | US | 68 | 24 |  | screen time | 7 | 2 | 18 | 0 | 0 | 0 | 0 |
| Efstathiou (2016) | Greece | 729 | 20 |  |  | 1 | 8 | 10 | 1 | 0 | 0 | 1 |
| Epstein (2004) | US | 63 | 24 | tv/VCR/DVDs, videogames, computer for non-school activities | sedentary time | 11 | 8 | 12 | 1 | 0 | 0 | 1 |
| Escobar-Chaves (2012) | US | 202 | 24 | tv | tv/dvd/vidoes | 12 | 6 | 9 | 1 | 0 | 0 | 1 |
| Essery (2008) | US | 92 | 12 |  | screen time | 5 | 2 | 5 | 1 | 0 | 0 | 1 |
| Ezendam (2012) | Netherlands | 883 | 10 |  | screen time |  | 12 | 13 | 1 | 0 | 0 | 1 |
| Faghy (2021) | UK | 147 | 12 |  | screen time | 2 | 7 | 11 | 0 | 0 | 0 | 0 |
| Fassnacht (2014) | Portugal | 49 | 8 |  | screen time | 8 | 8 | 10 | 1 | 0 | 0 | 1 |
| Fernandes (2016) | Brazil | 215 | 36 |  | screen time | 3 | 7 | 10 | 0 | 0 | 0 | 0 |
| Filho (2019) | Brazil | 1272 | 16 |  | tv/dvd/vidoes | 5 | 11 | 18 | 1 | 0 | 0 | 1 |
| Fitzgibbon (2005) | US | 409 | 14 |  | tv/dvd/vidoes | 6 | 3 | 5 | 1 | 0 | 0 | 1 |
| Fitzgibbon (2006) | US | 401 | 14 |  | tv/dvd/vidoes | 4 | 3 | 5 | 1 | 0 | 0 | 1 |
| Fitzgibbon (2011) | US | 618 | 14 | tv | tv/dvd/vidoes | 7 | 3 | 5 | 1 | 0 | 0 | 1 |
| Fitzgibbon (2013) | US | 147 | 14 | tv | tv/dvd/vidoes | 8 | 3 | 5 | 1 | 0 | 0 | 1 |
| Folta (2013) | US | 963 | 104 |  | tv/dvd/vidoes | 9 | 6 | 8 | 0 | 0 | 0 | 0 |
| Ford (2002) | US | 28 | 4 | tv, videogames | screen time | 9 | 7 | 12 | 1 | 1 | 1 | 3 |
| Foster (2008) | US | 1349 | 104 | tv, videogames | tv/dvd/vidoes | 12 | 10 | 12 | 1 | 0 | 0 | 1 |
| French (2011) | US | 90 | 52 | tv | tv/dvd/vidoes | 14 | 12 | 17 | 1 | 0 | 0 | 1 |
| French (2016) | US | 40 | 24 | tv, phone | tv/dvd/vidoes | 6 | 5 | 12 | 1 | 0 | 0 | 1 |
| French (2018) | US | 534 | 156 |  | screen time | 3 | 2 | 4 | 1 | 0 | 1 | 2 |
| Fulkerson (2018) | US | 160 | 40 |  | screen time | 17 | 8 | 12 | 1 | 0 | 0 | 1 |
| Gentile (2009) | US | 1323 | 32 | tv, videogames | screen time | 7 | 8 | 10 | 1 | 0 | 0 | 1 |
| Golan (1998) | Israel | 60 | 52 |  | tv/dvd/vidoes | 7 | 6 | 11 | 1 | 0 | 0 | 1 |
| Golan (2006) | Israel | 32 | 24 |  |  | 9 | 6 | 11 | 1 | 1 | 1 | 3 |
| Gonzalez-Jiminez (2013) | Spain | 138 | 34 |  |  | 1 | 14 | 19 | 0 | 0 | 0 | 0 |
| Gorin (2006) | US | 6 | 8 | tv | tv/dvd/vidoes | 6 | 2 | 14 | 0 | 0 | 0 | 0 |
| Gortmaker (1999) - Impact of | US | 785 | 104 | tv |  | 3 | 9 | 9 | 0 | 0 | 0 | 0 |
| Gortmaker (1999) - Reducing Obesity | US | 1560 | 104 | tv |  | 9 | 11 | 12 | 1 | 0 | 0 | 1 |
| Grydeland (2013) | Norway | 700 | 80 | tv/DVD, computer games | sedentary time | 7 | 11 | 12 | 1 | 0 | 0 | 1 |
| Haines (2013) | US | 121 | 24 | tv | tv/dvd/vidoes | 15 | 2 | 5 | 1 | 1 | 0 | 2 |
| Haire-Joshu (2021) | US | 85 | 24 |  | sedentary time | 12 | 2 | 17 | 0 | 0 | 0 | 0 |
| Hammersley (2019) | Australia | 86 | 11 |  | screen time | 11 | 2 | 5 | 1 | 0 | 1 | 2 |
| Handel (2017) | Denmark | 3058 | 60 | tv | screen time | 6 | 2 | 6 | 1 | 0 | 0 | 1 |
| Harrison (2006) | Ireland | 312 | 16 | tv, computer | screen time | 8 | 9 | 11 | 1 | 0 | 0 | 1 |
| Hidayanty (2016) | Indonesia | 238 | 12 |  | sedentary time | 13 | 11 | 15 | 1 | 0 | 1 | 2 |
| Hinkley (2015) | Australia | 22 | 5 | tv/DVD/video, computer, sedentary electronic games | screen time | 14 | 2 | 3 | 1 | 1 | 1 | 3 |
| Hull (2018) | US | 272 | 52 |  | screen time | 5 | 5 | 7 | 1 | 1 | 0 | 2 |
| Jacobs (2013) | South Africa | 325 | 36 |  | screen time | 4 | 10 | 11 | 0 | 0 | 0 | 0 |
| Jamerson (2017) | US | 3813 | 28 |  | tv/dvd/vidoes | 6 | 10 | 12 | 0 | 0 | 0 | 0 |
| Johnson (2005) | US | 10204 | 24 | tv | tv/dvd/vidoes | 1 | 1 | 5 | 0 | 0 | 0 | 0 |
| Johnston (2006) | US | 439 | 156 |  | tv/dvd/vidoes | 3 | 0 | 0 | 1 | 0 | 0 | 1 |
| Jones (2008) | US | 718 | 72 |  | tv/dvd/vidoes | 8 | 11 | 12 | 1 | 0 | 0 | 1 |
| Kameswararao (2009) | India | 350 | 24 |  | tv/dvd/vidoes | 6 | 6 | 14 | 0 | 0 | 0 | 0 |
| Keita (2014) | US | 50 | 20 | tv | tv/dvd/vidoes | 14 | 2 | 5 | 0 | 0 | 0 | 0 |
| Kelder (2004) | US | 258 | 20 |  | tv/dvd/vidoes | 8 | 9 | 9 | 0 | 0 | 0 | 0 |
| Killough (2010) | Canada | 35 | 12 |  |  | 6 | 9 | 11 | 0 | 0 | 0 | 0 |
| King (2013) | US | 3385 | 156 |  |  | 6 | 7 | 13 | 0 | 0 | 0 | 0 |
| Kipping (2008) | England | 679 | 20 | tv | screen time | 8 | 9 | 10 | 1 | 1 | 1 | 3 |
| Kipping (2014) | England | 2221 | 28 | tv | screen time | 6 | 9 | 10 | 1 | 1 | 1 | 3 |
| Knowlden (2015) | US | 57 | 4 | tv, computer, portable electronic device | screen time | 11 | 4 | 6 | 1 | 0 | 1 | 2 |
| Kobel (2014) | Germany | 1943 | 52 |  | screen time | 5 | 6 | 7 | 1 | 0 | 0 | 1 |
| Kobel (2020) | Germany | 154 | 36 |  | screen time | 6 | 5 | 8 | 1 | 0 | 0 | 1 |
| Kocken (2016) | Netherlands | 863 | 16 |  | sedentary time | 6 | 9 | 11 | 1 | 0 | 0 | 1 |
| Kong (2010) | US | 28 | 10 | tv | tv/dvd/vidoes | 4 | 5 | 11 | 0 | 0 | 0 | 0 |
| Kong (2016) | US | 618 | 14 | tv | tv/dvd/vidoes | 5 | 3 | 5 | 1 | 0 | 0 | 1 |
| Leme (2016) | Brazil | 253 | 24 |  | tv/dvd/vidoes | 11 | 14 | 18 | 1 | 0 | 1 | 2 |
| Lerner-Geva (2015) | Israel | 204 | 16 |  | screen time | 5 | 4 | 6 | 1 | 0 | 1 | 2 |
| Li (2019) | China | 1641 | 52 |  | screen time | 11 | 6 | 7 | 1 | 0 | 1 | 2 |
| Lin (2021) | Taiwan | 129 | 8 |  | screen time | 16 | 4 | 6 | 1 | 1 | 1 | 3 |
| Ling (2018) | US | 73 | 10 |  | screen time | 16 | 3 | 5 | 1 | 0 | 0 | 1 |
| Liu (2019) | China | 1839 | 8 |  | sedentary time | 9 | 7 | 11 | 1 | 0 | 0 | 1 |
| Lubans (2008) | Australia | 116 | 8 | tv, computer, videogames | tv/dvd/vidoes | 12 | 14 | 15 | 0 | 0 | 0 | 0 |
| Lubans (2009) | Australia | 124 | 24 | tv, computer, electronic games | tv/dvd/vidoes | 11 | 14 | 15 | 1 | 0 | 0 | 1 |
| Lubans (2012) | Australia | 357 | 52 |  | screen time | 11 | 14 | 15 | 1 | 0 | 1 | 2 |
| Lubans (2016) | Australia | 361 | 20 |  | screen time | 7 | 12 | 14 | 1 | 1 | 0 | 2 |
| Lumeng (2017) | US | 697 | 52 |  | screen time | 10 | 3 | 5 | 1 | 0 | 0 | 1 |
| Lynch (2016) | US | 51 | 16 | tv | tv/dvd/vidoes | 3 | 7 | 8 | 1 | 0 | 0 | 1 |
| Maddison (2014) | New Zealand | 251 | 20 | tv, computer | sedentary time | 11 | 9 | 12 | 1 | 1 | 0 | 2 |
| Marsh (2020) | New Zealand | 51 | 6 | tv, other screen devices | screen time | 8 | 2 | 4 | 1 | 1 | 0 | 2 |
| McGarvey (2004) | US | 336 | 52 | tv | tv/dvd/vidoes | 6 | 2 | 4 | 0 | 0 | 0 | 0 |
| Melero-Canas (2021) | Spain | 150 | 36 |  | sedentary time | 11 | 13 | 15 | 1 | 0 | 0 | 1 |
| Mendelsohn (2011) | US | 450 | 24 | tv | screen time | 7 | 0.42 | 0.67 | 1 | 1 | 1 | 3 |
| Mendoza (2016) | US | 160 | 8 | tv, computers, other electronic devices | tv/dvd/vidoes | 8 | 3 | 5 | 1 | 0 | 0 | 1 |
| Morgan (2019) | Australia | 153 | 8 |  | screen time | 16 | 4 | 12 | 1 | 0 | 0 | 1 |
| Moshki (2016) | Iran | 95 | 4 | tv | tv/dvd/vidoes | 4 | 6 | 10 | 1 | 0 | 0 | 1 |
| Mouttapa (2016) | US | 523 | 52 |  |  | 7 | 8 | 11 | 0 | 0 | 0 | 0 |
| Natale (2014) | US | 307 | 24 | tv | sedentary time | 6 | 2 | 5 | 1 | 0 | 0 | 1 |
| Neumark-Sztainer (2010) | US | 356 | 16 |  | tv/dvd/vidoes | 12 | 14 | 16 | 1 | 0 | 0 | 1 |
| Ni Mhurchu (2009) | New Zealand | 29 | 6 | tv | tv/dvd/vidoes | 4 | 9 | 12 | 1 | 0 | 0 | 1 |
| Nosi (2021) | Italy | 863 | 32 |  | screen time | 8 |  |  | 1 | 0 | 0 | 1 |
| Novotny (2018) | US | 4333 | 104 |  | screen time | 6 | 2 | 8 | 1 | 0 | 0 | 1 |
| Nyberg (2015) | Sweden | 243 | 24 |  | screen time | 12 | 6 | 6 | 1 | 0 | 0 | 1 |
| Nyberg (2016) | Sweden | 378 | 24 |  | screen time | 12 | 6 | 6 | 1 | 0 | 0 | 1 |
| O'Dwyer (2012) | England | 79 | 10 |  | sedentary time | 11 | 3 | 4.9 | 1 | 0 | 0 | 1 |
| Ostbye (2012) | US | 400 | 32 |  | tv/dvd/vidoes | 11 | 2 | 5 | 1 | 0 | 0 | 1 |
| Paradis (2005) | Canada | 657 | 104 |  | tv/dvd/vidoes | 2 | 6 | 11 | 0 | 0 | 0 | 0 |
| Pathirana (2018) | Australia | 640 | 8 |  | screen time | 5 | 0 | 5 | 0 | 0 | 0 | 0 |
| Patrick (2006) | US | 819 | 52 |  | tv/dvd/vidoes | 13 | 11 | 15 | 1 | 0 | 0 | 1 |
| Pbert (2016) | US | 37 | 6 |  | computer | 13 | 8 | 12 | 0 | 0 | 0 | 0 |
| Pearson (2020) | UK | 75 | 12 |  | tv/dvd/vidoes | 15 | 9 | 11 | 1 | 0 | 0 | 1 |
| Peralta (2009) | Australia | 33 | 24 |  | screen time | 9 | 12 | 13 | 1 | 0 | 1 | 2 |
| Peterson (2015) | US | 45 | 156 | tv | tv/dvd/vidoes | 1 | 12 | 12 | 0 | 0 | 0 | 0 |
| Plachta-Danielzik (2007) | Germany | 1764 | 3 | tv | screen time | 4 | 10 | 10 | 1 | 0 | 0 | 1 |
| Plachta-Danielzik (2011) | Germany | 1192 | 3 | tv | screen time | 4 | 6 | 6 | 1 | 0 | 0 | 1 |
| Puder (2011) | Switzerland | 652 | 36 | tv, computer, videogames | screen time | 8 | 4 | 5 | 1 | 0 | 1 | 2 |
| Ribiero (2014) | Brazil | 2038 | 28 | tv/DVD, computer/videogames | tv/dvd/vidoes | 2 | 6 | 11 | 1 | 0 | 0 | 1 |
| Rito (2013) | Portugal | 266 | 24 | tv | screen time | 8 | 6 | 10 | 0 | 0 | 0 | 0 |
| Robinson (1999) | US | 227 | 24 | tv, video, videogames | screen time | 8 | 8 | 9 | 1 | 0 | 1 | 2 |
| Robinson (2003) | US | 61 | 12 | tv, video, videogames | tv/dvd/vidoes | 9 | 8 | 10 | 1 | 0 | 1 | 2 |
| Robinson (2006) | US | 225 | 24 | tv, video, videogames | computer | 18 | 8 | 9 | 1 | 0 | 1 | 2 |
| Robinson (2010) | US | 284 | 104 | tv, video, videogames, computer | tv/dvd/vidoes | 5 | 8 | 10 | 1 | 0 | 0 | 1 |
| Romo (2018) | Ecuador | 307 | 28 |  | screen time | 9 | 3 | 4 | 0 | 0 | 0 | 0 |
| Sacher (2010) | UK | 116 | 24 |  | sedentary time | 8 | 8 | 12 | 1 | 0 | 0 | 1 |
| Saelens (2002) | US | 44 | 16 |  | sedentary time | 13 | 12 | 16 | 1 | 1 | 0 | 2 |
| Sahota (2001) | UK | 636 | 52 |  | sedentary time | 1 | 7 | 11 | 1 | 0 | 0 | 1 |
| Salmon (2006) | Australia | 164 | 36 | tv | tv/dvd/vidoes | 6 | 10 | 10 | 1 | 0 | 0 | 1 |
| Salmon (2008) | Australia | 311 | 36 | tv | tv/dvd/vidoes | 11 | 10 | 11 | 1 | 0 | 0 | 1 |
| Salmon (2011) | Australia | 1048 | 7 | tv, computer | tv/dvd/vidoes | 5 | 9 | 12 | 1 | 0 | 0 | 1 |
| Sanders (2019) | US | 39 | 0.0119 |  |  | 6 | 5 | 12 | 1 | 0 | 0 | 1 |
| Santina (2021) | Lebanon | 374 | 14 |  | screen time | 13 | 10 | 12 | 1 | 1 | 1 | 3 |
| Schwartz (2012) | US | 59 | 12 |  |  | 3 | 6 | 11 | 0 | 0 | 0 | 0 |
| Serra-Paya (2014) | Spain | 86 | 36 |  | screen time | 7 | 8 | 12 | 0 | 0 | 0 | 0 |
| Shelton (2007) | Australia | 43 | 4 |  | screen time | 8 | 3 | 10 | 1 | 0 | 1 | 2 |
| Shrewsbury (2020) | Australia | 3203 | 13 |  | screen time | 9 | 13 | 14 | 0 | 0 | 0 | 0 |
| Simon (2008) | France | 954 | 208 |  | tv/dvd/vidoes | 4 | 9 | 13 | 1 | 0 | 0 | 1 |
| Singh (2009) | Netherlands | 1108 | 32 |  | screen time | 1 | 12 | 14 | 1 | 0 | 1 | 2 |
| Singhal (2010) | India | 209 | 24 | tv | tv/dvd/vidoes | 10 | 15 | 17 | 1 | 0 | 0 | 1 |
| Skouteris (2016) | Australia | 201 | 10 |  | screen time | 7 | 2 | 4 | 1 | 1 | 1 | 3 |
| Slootmaker (2010) | Netherlands | 87 | 12 |  | sedentary time | 4 | 13 | 17 | 1 | 0 | 0 | 1 |
| Smith (2014) | Australia | 361 | 20 |  | screen time | 13 | 12 | 14 | 1 | 0 | 1 | 2 |
| Spruit-Metz (2008) | US | 459 | 1 | tv, computer |  | 4 | 11 | 13 | 1 | 0 | 0 | 1 |
| St. George (2013) | US | 73 | 6 |  |  | 8 | 11 | 15 | 1 | 0 | 1 | 2 |
| Story (2003) | US | 54 | 12 | tv | tv/dvd/vidoes | 13 | 8 | 10 | 1 | 0 | 0 | 1 |
| Taveras (2011) - First Steps | US | 84 | 24 | tv | tv/dvd/vidoes | 11 | 0 | 0.5 | 0 | 0 | 0 | 0 |
| Taveras (2011) - RCT | US | 475 | 104 | tv | tv/dvd/vidoes | 9 | 2 | 6 | 1 | 0 | 0 | 1 |
| Taylor (2006) | New Zealand | 594 | 52 | tv | sedentary time | 3 | 5 | 12 | 0 | 0 | 0 | 0 |
| Todd (2008) | US | 22 | 20 | tv, movie/video, computer/internet for non-school purposes, videogames | screen time | 7 | 8 | 11 | 1 | 0 | 1 | 2 |
| Tomayko (2016) | US | 150 | 104 | tv | tv/dvd/vidoes | 1 | 2 | 5 | 1 | 0 | 1 | 2 |
| Trost (2009) | US | 105 | 4 |  | screen time | 3 | 6 | 10 | 1 | 0 | 0 | 1 |
| Tucker (2013) | US | 125 | 24 | tv, computer | tv/dvd/vidoes | 6 | 4 | 18 | 1 | 0 | 0 | 1 |
| Tuominen (2017) | Finland | 228 | 8 |  | screen time | 3 | 5 | 7 | 1 | 0 | 0 | 1 |
| Van Grieken (2014) | Netherlands | 737 | 104 | tv | tv/dvd/vidoes | 2 | 5 | 5 | 1 | 0 | 0 | 1 |
| Van Stralen (2012) | Netherlands | 708 | 68 |  | tv/dvd/vidoes | 10 | 8 | 12 | 0 | 0 | 0 | 0 |
| Verbestel (2013) | Belgium | 203 | 52 |  | screen time | 4 | 0.75 | 2 | 1 | 0 | 0 | 1 |
| Verloigne (2012) | Belgium | 740 | 6 |  | sedentary time | 11 | 10 | 12 | 1 | 0 | 0 | 1 |
| Vik (2015) | Belgium, Germany, Greece, Hungary, Norway | 3394 | 6 |  | tv/dvd/vidoes | 11 | 10 | 12 | 1 | 0 | 0 | 1 |
| Vlasblom (2020) | Netherlands | 1521 | 156 | tv, computer | screen time | 15 | 0 | 3 | 1 | 0 | 0 | 1 |
| Wadolowska (2019) | Poland | 464 | 3 |  | screen time | 2 | 11 | 12 | 0 | 0 | 0 | 0 |
| Walton (2015) | Canada | 48 | 9 |  |  | 8 | 2 | 5 | 1 | 1 | 0 | 2 |
| Weber (2014) | Australia | 64 | 10 | tv | screen time | 2 | 0 | 5 | 0 | 0 | 0 | 0 |
| Wen (2020) | Australia | 1155 | 104 |  | screen time | 12 | 0 | 0.83 | 1 | 1 | 1 | 3 |
| Whaley (2010) | US | 821 | 52 | tv | tv/dvd/vidoes | 3 | 1 | 5 | 0 | 0 | 1 | 1 |
| Whittemore (2013) | US | 384 | 24 |  | sedentary time | 12 | 14 | 17 | 0 | 0 | 0 | 0 |
| Williamson (2007) | US | 661 | 104 | tv | tv/dvd/vidoes | 3 | 5 | 13 | 1 | 0 | 0 | 1 |
| Willis (2014) | England | 77 | 8 | tv | screen time | 2 | 3 | 7 | 0 | 0 | 0 | 0 |
| Willis (2016) | UK | 624 | 8 | tv | screen time | 2 | 0 | 5 | 0 | 0 | 1 | 1 |
| Wofford (2013) | US | 46 | 12 |  | screen time | 3 | 6 | 14 | 0 | 0 | 0 | 0 |
| Xu (2015) | China | 1182 | 36 |  | screen time | 2 | 9 | 10 | 1 | 0 | 0 | 1 |
| Yoshinaga (2020) | Japan | 156 | 12 | ST (tv, electronic games on tv, computer, cell phone or other devices) | screen time | 5 | 6 | 12 | 1 | 1 | 1 | 3 |
| Zimmerman (2012) | US | 67 | 16 | tv | tv/dvd/vidoes | 6 | 2.5 | 4.5 | 1 | 0 | 0 | 1 |
