# Supplementary material for: Identifying effective intervention strategies to reduce children’s screen time: a systematic review and meta-analysis
Source: Int J Behav Nutr Phys Act. 2021 Sep 16;18:126. doi: 10.1186/s12966-021-01189-6 (PMC8447784; doi:10.1186/s12966-021-01189-6)
Supplement: Supplementary file 5 [file 12966_2021_1189_MOESM5_ESM.docx]

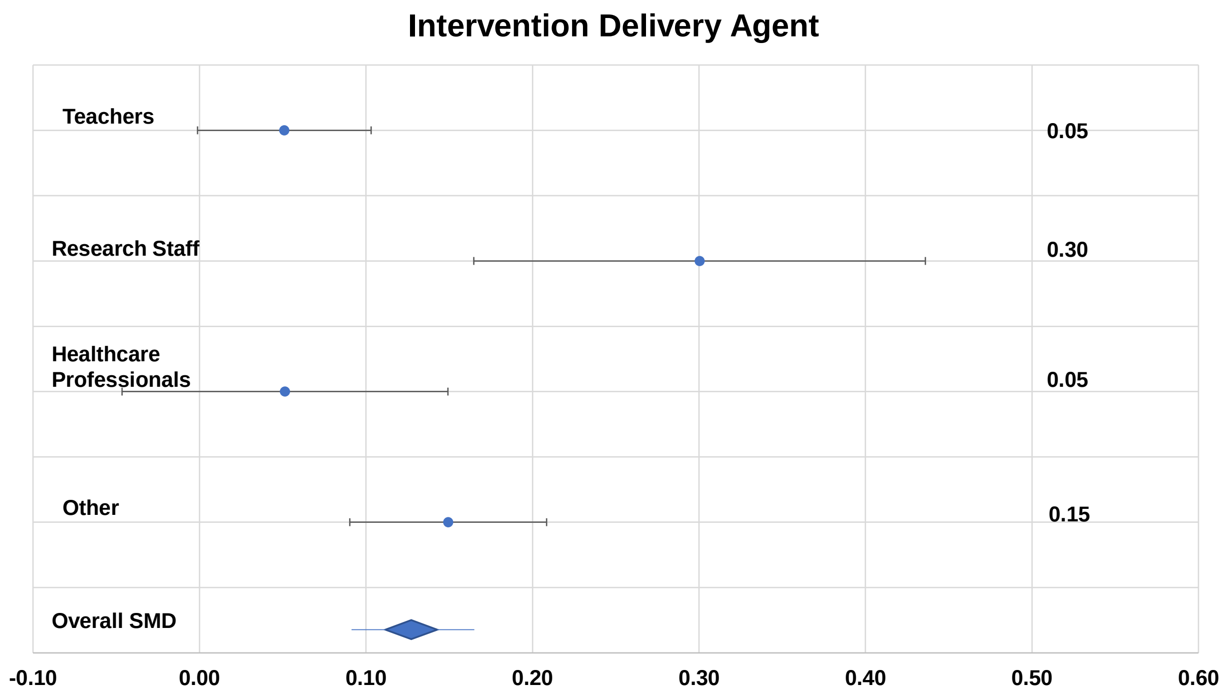
Intervention Delivery Agent
Teachers
0.05
Research Staff
0.30
Healthcare Professionals
0.05
Other
0.15
Overall SMD
-0.10
0.00
0.10
0.20
0.30
0.40
0.50
0.60

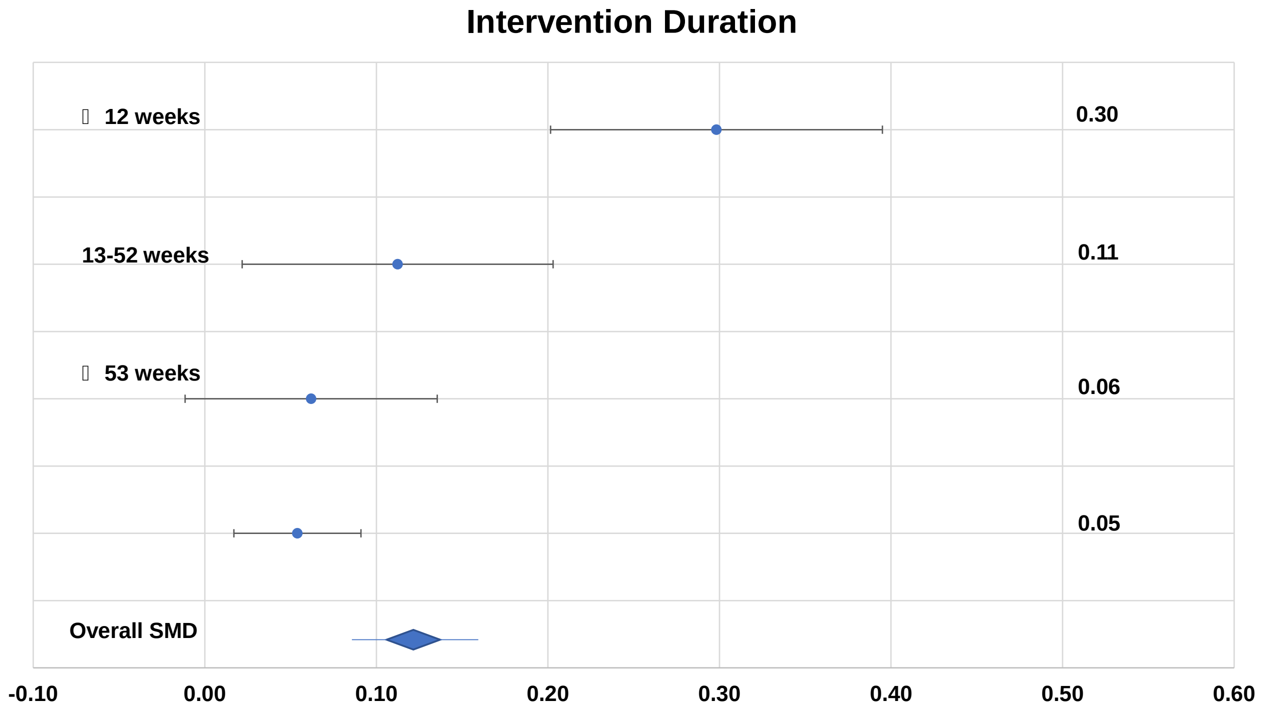
Intervention Duration
12 weeks
0.30
13-52 weeks
0.11
53 weeks
0.06
0.05
Overall SMD
-0.10
0.00
0.10
0.20
0.30
0.40
0.50
0.60

Sample Size

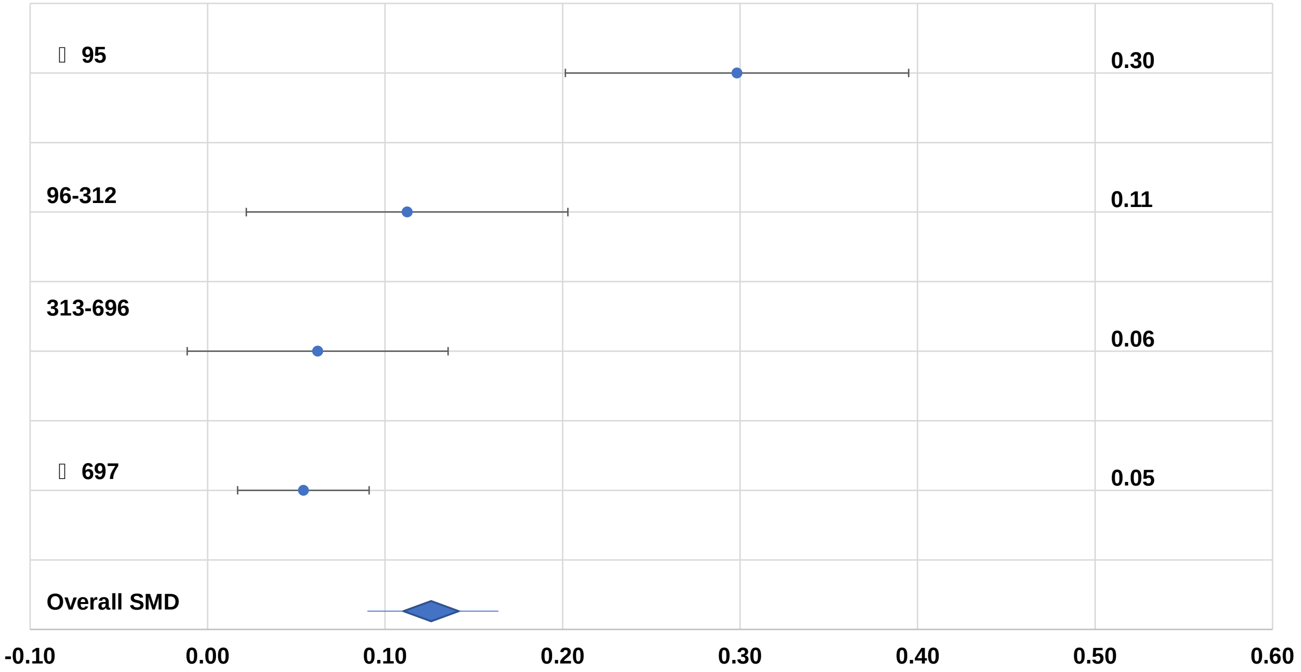
95
0.30
96-312
0.11
313-696
0.06
697
0.05
Overall SMD
-0.10
0.00
0.10
0.20
0.30
0.40
0.50
0.60
